# The PAD4 inhibitor GSK484 diminishes neutrophil extracellular trap in the colon mucosa but fails to improve inflammatory biomarkers in experimental colitis

**DOI:** 10.1042/BSR20253205

**Published:** 2025-06-11

**Authors:** Kangzhe Xie, Jordan Hunter, Aaron Lee, Gulfam Ahmad, Paul K. Witting, Tamara Ortiz-Cerda

**Affiliations:** 1 Redox Biology Group, School of Medical Sciences, Faculty of Medicine & Health, The University of Sydney, Sydney, New South Wales 2006, Australia; 2 Charles Perkins Centre, School of Medical Sciences, Faculty of Medicine & Health, The University of Sydney, Sydney, New South Wales 2006, Australia; 3 Department of Biological Sciences, Purdue University, West Lafayette, Indiana, U.S.A.; 4 Andrology Department, Royal Women’s and Children’s Pathology, Carlton, Victoria 3053, Australia; 5 Departmento de Citología e Histología Normal y patológica, Facultad de Medicina, Universidad de Sevilla, Avda. Sánchez-Pizjuán s/n Sevilla, Seville 41009, Spain

**Keywords:** inflammatory bowel disease, mast cells, NET, neutrophils, ulcerative colitis

## Abstract

Inflammatory bowel disease (IBD) is a gastrointestinal disorder characterised by elevated colonic neutrophil extracellular traps (NETs), which are associated with disease severity. Formation of NETs is primarily driven by peptidyl arginine deaminase IV (PAD4) and other enzymes including myeloperoxidase (MPO) and neutrophil elastase. The present study evaluated the effect of MPO and PAD4 inhibition in dextran sodium sulfate (DSS)-induced colitis. Experimental colitis was induced in male C57BL/6 mice by 2% w/v DSS in drinking water ad libitum. Treatment groups received daily oral administration of MPO inhibitor (AZD3241; 30 mg/kg) and/or intraperitoneal injection of PAD4 inhibitor (GSK484; 4 mg/kg) 4 times over 9 days. Inhibition of PAD4 significantly diminished NET density in the colonic mucosa of mice insulted with DSS, reaching levels similar to that detected in control mice. Both inhibitors offered limited improvement in disease-activity-index, a scoring system that considers the extent of weight loss, stool consistency and rectal bleeding. Histology showed that MPO and/or PAD4 inhibition did not recover DSS-induced colon histoarchitectural damage whilst Alcian blue staining demonstrated that PAD4 failed to reduce goblet cell loss. The selected dosage of PAD4 inhibition also yielded no effect on inflammatory markers and antioxidant protein levels. These data sets suggest that other mechanisms may be involved in the pathogenesis of IBD, and the appropriate dosage of GSK484 requires thorough investigation.

## Introduction

Inflammatory bowel disease (IBD) is an umbrella term that encapsulates Crohn’s disease (CD) and ulcerative colitis (UC). These chronic autoimmune conditions manifest as severe gastrointestinal disorders, predominantly confined in the colon in UC or affecting any part of the digestive tract in CD [1]. The general clinical symptoms of IBD include abdominal pain, weight loss, diarrhoea, rectal bleeding, fever and anaemia [2,3]; all factors that affect lifestyle and clinical outcomes in patients diagnosed with IBD [4]. Currently, there is no cure for IBD and contemporary treatments such as 5-aminosalicylates, corticosteroids, immunomodulators and biologic therapies (e.g. anti-tumour necrosis factor alpha [TNF-α]) [5] all address symptoms. However, patients often experience disease relapse with adverse side effects such as nausea, vomiting and compromised immune systems [6].

The cause of IBD is largely unknown; however, risk factors such as age, familial history, ethnicity and environmental factors (e.g. diet and smoking), use of antibiotics and non-steroidal anti-inflammatory drugs are all linked to IBD pathogenesis [7]. Specifically, UC is characterised by immune cell infiltration, namely mast cells (MC) and neutrophils into the colon epithelium. This characteristic immune infiltration correlates with an elevation of calprotectin (CP; from activated neutrophils) level in the stool [8]. However, the underlying mechanism driving immune recruitment remains elusive. The current dogma identifies invading pathogenic bacteria, erosion of the colon epithelium and formation of colonic lesions in the presence of reactive oxygen species (ROS) as potentiating factors [9].

Increased MC infiltration and activation in the ileum and colon of IBD patients has been demonstrated [10-12]. Similarly, MC infiltration and activation is also described in animal models of UC and CD [13,14]. Upon activation by interleukin (IL)-18, tissue-resident MC degranulate to release inflammatory mediators including histamine and serine proteases [15]. In parallel, activated MCs interact with dendritic cells to secrete pro-inflammatory cytokines interferon (IFN)-γ and IL-17, which together promote T cell differentiation to yield Th1 and Th17 phenotypes [16]. Activated neutrophils have been reported to cross-talk with the IL-18/IL-18 receptor (IL-18R)-Th1 polarisation signalling pathway, where IL-18R stimulation on natural killer and Th1 cells results in neutrophil recruitment to the site of inflammation [17]. Furthermore, the G-protein-coupled receptor GPR35 contributes to efficient recruitment of neutrophils *in vivo*, where the MC-derived serotonin metabolite, 5-hydroxyindoleacetic acid (5-HIAA), acts as ligand for this receptor [18], suggesting a direct interplay between MCs and neutrophils during the inflammatory response.

Myeloperoxidase (MPO) is a heme enzyme that is abundantly found in the lysosomal azurophilic granules of neutrophils, comprising ~5% of the neutrophil dry mass [19]. Enzymic MPO utilises hydrogen peroxide (H_2_O_2_) and chloride anions (Cl^−^) as substrates, catalysing the production of hypochlorous acid (HOCl) [20]. The potent cytotoxic oxidant HOCl elicits bactericidal actions and eliminates invading pathogens by inducing non-specific DNA damage [21]. However, the release of neutrophil MPO into the extracellular space can also cause host tissue damage. Together, infiltrating immune cells and host tissue damage become central to IBD pathogenesis that results in further pathogenic bacterial invasion and cyclic recruitment of immune cells to the inflamed colon.

The immune system tightly regulates the action of neutrophils via degranulation, phagocytosis and the formation of neutrophil extracellular traps (NETs) [22], which are extracellular structures that contain cytosolic and granule proteins (including MPO) [23]. Formation of NETs (NETosis) is initiated by nuclear chromatin decondensation, where ROS drive the translocation of neutrophil elastase (NE) to the nucleus, disrupting the chromatin structure [24]. This nuclear disruption is followed by the binding of MPO to chromatin and the conversion of arginine residues into citrullinated histone 3 (citH3) by peptidyl-arginine deiminase IV (PAD4), stimulating the disassembly of the nuclear envelope [23,25]. Nuclear envelope disassembly initiates the rupturing of the neutrophil membrane, releasing the cytosolic and granule contents into the extracellular space, which enables sustained bactericidal effects to be observed after cell death [26].

The dysregulation of NETs has been identified in various chronic inflammatory conditions. For example, increased NET density is detected in the synovial fluid of patients with rheumatoid arthritis [27], and patients diagnosed with chronic obstructive pulmonary disease or small vessel vasculitis [28]. Recently, the association of dysregulated NETs formation and IBD has been established [29-31], whereby elevated NETs density was observed in patients with CD and UC when compared with healthy controls. Furthermore, through the utilisation of multiplex imaging of NE, MPO and citH3, Schroder et al. showed that an increasing colonic NET density correlates with greater disease severity in CD [32], highlighting the potential involvement of NETs in the pathogenesis of IBD.

Pharmacological inhibition of MPO by synthetic inhibitor AZD3241 has shown to improve experimental colitis symptoms and activate the heme oxygenase-1 (HO-1)/nuclear factor erythroid factor 2-related factor 2 (Nrf2) signalling pathways [33], whilst the inhibition of PAD4 by the pan-PAD inhibitor Cl-Amidine ameliorated experimental colitis and up-regulated glutathione peroxidase 1 (GPx1) and superoxide dismutase 1 (SOD1) expression [34,35]. However, the exact involvement of NETs in experimental IBD pathogenesis remains to be fully defined, as the spatial co-localisation of essential markers of NETs: MPO, NE and citH3 was not examined. Herein, we examined the therapeutic potential of two selective enzyme inhibitors: AZD3241 (inhibiting MPO) and GSK484 (inhibiting PAD4) in ameliorating dextran sodium sulphate (DSS)-induced experimental colitis.

## Results

### Electrospray of MedChemExpress supplied AZD3241 contains impurities

Electrospray mass spectrometry analysis of sourced AZD3241 (*M* = 253.32 g/mol) obtained from Pharmaxis and MedChemExpress showed a high abundance peak at 254.09 m/z, indicating the detection of parent ion *M* + 1 (*M* + H) under positive ion mode. Compared with authentic AZD3241 supplied by Pharmaxis, the peak response at 254.09 m/z for the compound obtained from MedChemExpress was substantially lower. Additionally, a weak complex peak response was observed at 74.06 m/z (Supplementary Figure S1a-c), which suggests that AZD3241 sourced from MedChemExpress contained low-molecular weight contaminants (likely inorganic salts). Ratio difference calculation between the two MPO inhibitors showed that AZD3241 from MedChemExpress contained ~42% of the compound of interest relative to the same weight of inhibitor supplied by Pharmaxis. Thus, a compensatory dosage equivalent to 30 mg/kg for AZD3241 sourced from MedChemExpress was prepared prior to administration to mice in the current study.

### Administered MPO and/or PAD4 inhibitor failed to improve DSS-induced weight loss, disease activity index, nor alleviate macroscopic colon damage

**Figure 1:**
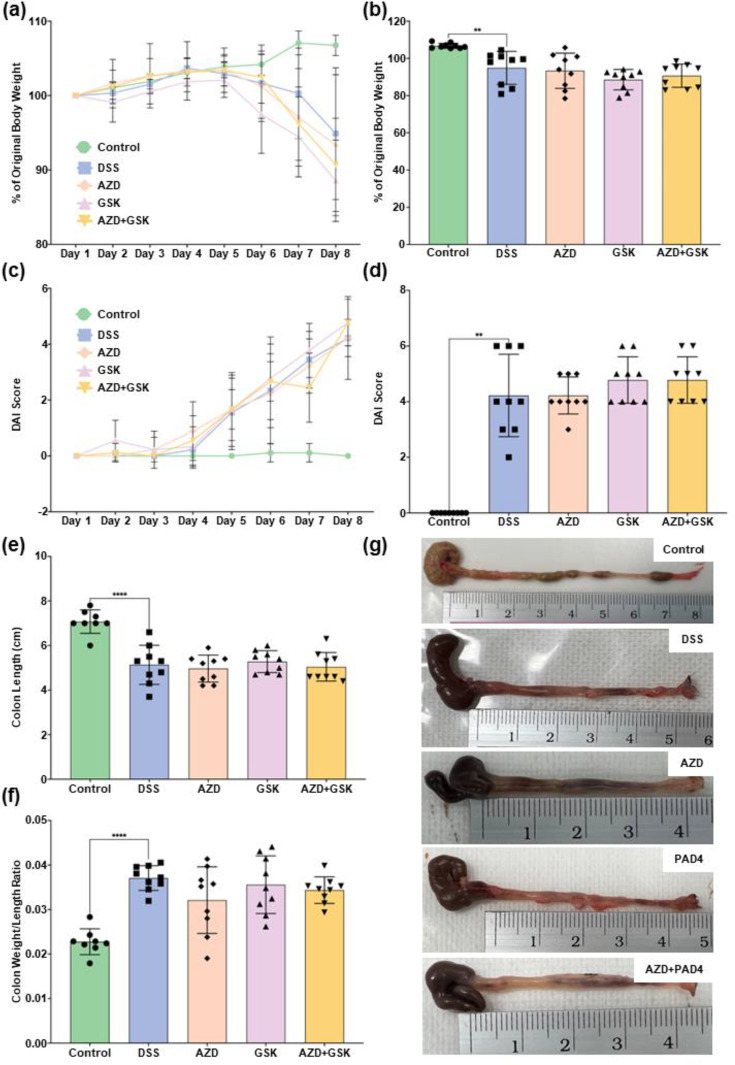
Effect of MPO and/or PAD4 inhibition on clinical outcomes and macroscopic colon damage in mice following eight days of DSS insult. (**a**) Percentage of the original weight from the start of the experiment to the day of sacrifice. (**b**) Percentage of original weight at the day of sacrifice. (**c**) DAI score recorded throughout the experiment period. (**d**) DAI score recorded at the day of sacrifice. (**e**) Isolated colon length at the day of sacrifice. (**f**) Isolated colon weight/length ratio recorded at the day of sacrifice. (**g**) Representative images of isolated colon from different experimental groups. Graphical values represent mean ± SD with *n* = 9 mice per group. Normalcy of the collected data was analysed using Shapiro–Wilk test, group difference was analysed by one way ANOVA with Tukey’s multiple comparison for parametric data and Kruskal–Wallis test with Dunn’s multiple comparison test was used for non-parametric data. *P≤0.05, ** *P*≤0.01, ***P≤0.001 and ****P≤0.0001. DAI, disease activity index; DSS, dextran sodium sulphate; MPO, myeloperoxidase; PAD4, peptidyl arginine deaminase IV.

DSS is a water-soluble polysaccharide that promotes gut epithelial monolayer damage when administered orally, which results in a loss of body weight and intestinal inflammation that mimics a UC-like condition in rodents [36]. Here, mice supplemented with DSS progressively lost weight from day 5, whilst control mice (absence of DSS or drug intervention) continued to increase body weight throughout the monitoring period (Figure 1a). At day 8 (day of sacrifice), mice insulted with DSS recorded a significant reduction in body weight when compared with the control (*P*=0.0066, Figure 1b). However, pharmacological inhibition with AZD, GSK or the combination of drugs (AZD+GSK group) was unable to mitigate DSS-induced weight loss. In mice supplemented with DSS, the disease activity index (DAI) scores increased markedly after day 4, yielding an average score of 4.5 at the end of monitoring (Figure 1c). By contrast, the DAI scores remained negligible in the control group over the same period. At the day of sacrifice, the DAI score from mice challenged with DSS was significantly greater than the corresponding control group (*P*=0.0087). In mice insulted with DSS in the presence of AZD3241 and/or GSK484, both inhibitors failed to ameliorate the DAI score (Figure 1d).

Next, we evaluated colon length and colon weight/length ratio as markers of macroscopic colon damage. As shown in Figure 1e and f, mice in the control group recorded a significantly longer colon length (*P*<0.0001) and lower colon weight/length ratio (*P*<0.0001) when compared with isolated colons from the DSS-insult group, whilst MPO and/or PAD4 inhibition did not improve these DSS-induced criteria. Representative images shown in Figure 1g demonstrated the dark-red colour of stool and reddened appearance of colons from DSS-insulted mice indicative of intestinal hyperaemia and colon inflammation, which was largely unaffected by any of the drug interventions. Indeed, determination of stool haemoglobin content demonstrated that DSS significantly increased faecal haemoglobin levels (*P*=0.0008) whilst MPO and PAD4 inhibitions had no effect (*P*>0.05, Supplementary Figure S2). Collectively, these outcomes demonstrate that insult with DSS resulted in extensive colon damage with parallel decline in colon function, whilst pharmacological inhibition of the enzymes MPO and/or PAD4 offered negligible protection to the colon.

### Pharmacologic inhibition of MPO and/or PAD4 did not reduce biomarkers of colonic inflammation

CP is a calcium-binding protein primarily produced by neutrophils, and elevated faecal CP (FCP) level directly reflects the extent of neutrophil infiltration in patients with IBD [37,38]. In the present study, significantly higher CP levels were observed in colon homogenates and stool samples from the mice that received DSS supplementation in their drinking water (*P*=0.0032 and *P*=0.038 respectively, Supplementary Figure S3a and b). The administration of AZD3241 or GSK484 failed to alleviate the elevated CP level in both colon tissue and faeces, suggesting that MPO or PAD4 inhibition did not reduce the extent of neutrophil recruitment to the inflamed colon.

### Colon damage elicited by DSS is not abrogated by MPO and/or PAD4 inhibition

**Figure 2:**
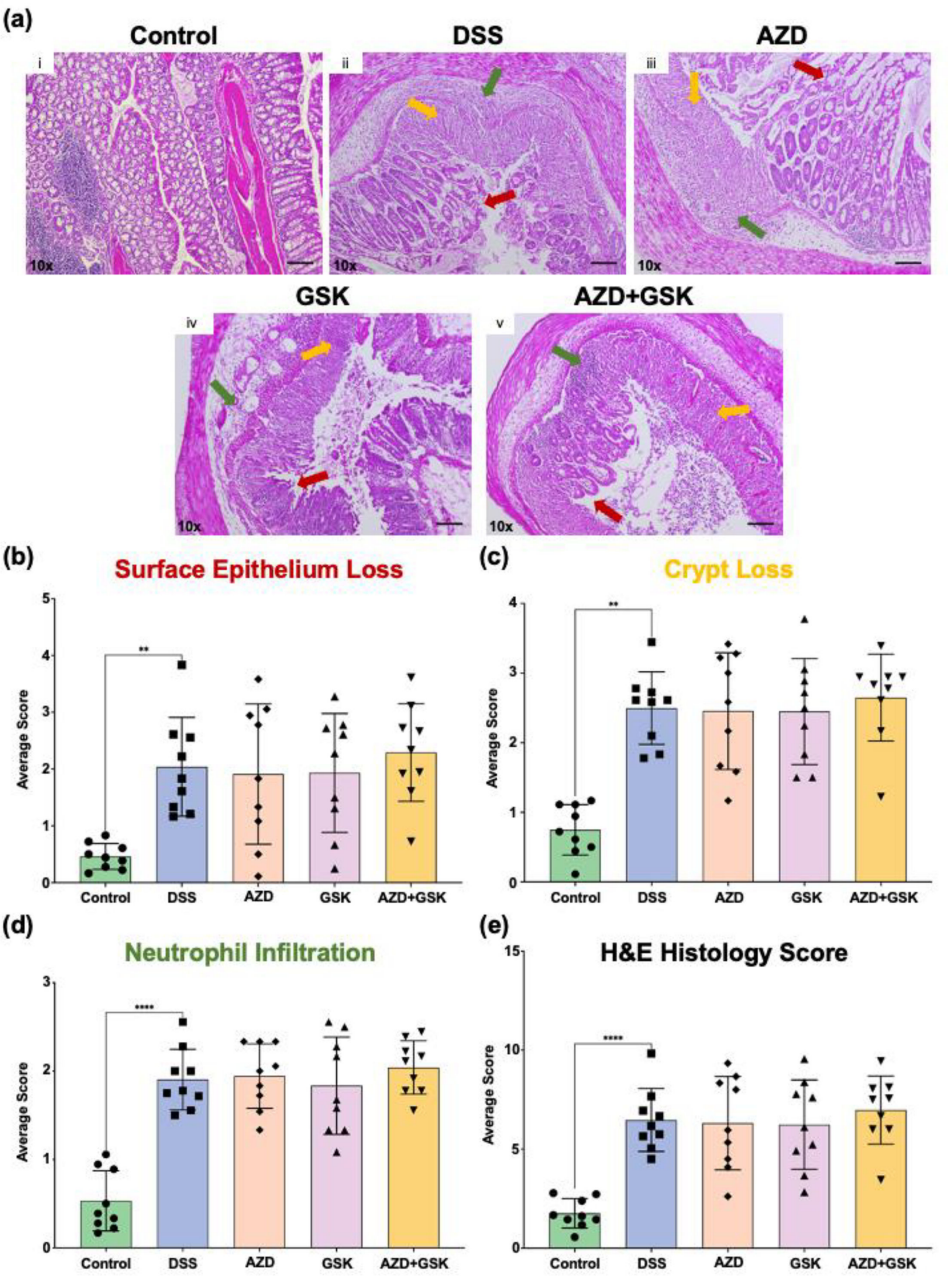
Haematoxylin and eosin (H&E) staining of isolated mouse colons and histopathological evaluation. (**a**) Representative colon images from different groups were captured from Axio Lab.A1 light microscope with a Axiocam 105 Color camera at 10 x magnification. (i–v) The type and location of histoarchitectural damage was highlighted: surface epithelium loss (red arrows), crypt loss (yellow arrows) and neutrophil infiltrations (green arrows). Scale bar = 100 µm. Histology score for (**b**) surface epithelium loss, (**c**) crypt loss, (**d**) neutrophil infiltration and (**e**) total histology score. Graphical values represent mean ± SD with *n* = 9 mice per group. Normalcy of the collect data was analysed using Shapiro–Wilk test, group difference was analysed by one way ANOVA with Tukey’s multiple comparison for parametric data and Kruskal–Wallis test with Dunn’s multiple comparison test was used for non-parametric data. *P≤0.05, ** *P*≤0.01, ***P≤0.001 and **** *P*≤0.0001.

The histoarchitecture of the isolated colons was visualised with H&E staining with a focus on surface epithelium loss, crypt loss and the extent of neutrophil infiltration. When compared with the control group, mice insulted with DSS exhibited significantly greater extent of surface epithelium loss, commonly showing severe erosion of the brush epithelial border (*P*=0.006, red arrows in Figure 2a(i,ii) and b). These tissue changes were accompanied by the presence of extensive colon ulceration, disruption of crypt histoarchitecture (*P*=0.0062, yellow arrows in Figure 2a(i,ii) and c) and pronounced oedema and neutrophil infiltration into the colon mucosa and submucosa (*P*<0.0001, green arrows in Figure 2a(i,ii) and d). Mice that received MPO and/or PAD4 inhibitors showed similar histopathologic characteristics in which AZD3241 and/or GSK484 administration failed to preserve colon histoarchitecture and reduce immune infiltration (Figure 2a(iii-v) and b-d).

As anticipated, combining the histological scoring showed that DSS insult caused extensive colon histoarchitectural disruption and immune infiltration, whereas pharmacological inhibition of MPO and/or PAD4 (either separately or in combination) was unable to ameliorate this DSS-induced colon damage (*P*<0.0001, Figure 2e).

### MPO and/or PAD4 inhibition did not prevent goblet cell loss nor mitigate mucin production

**Figure 3:**
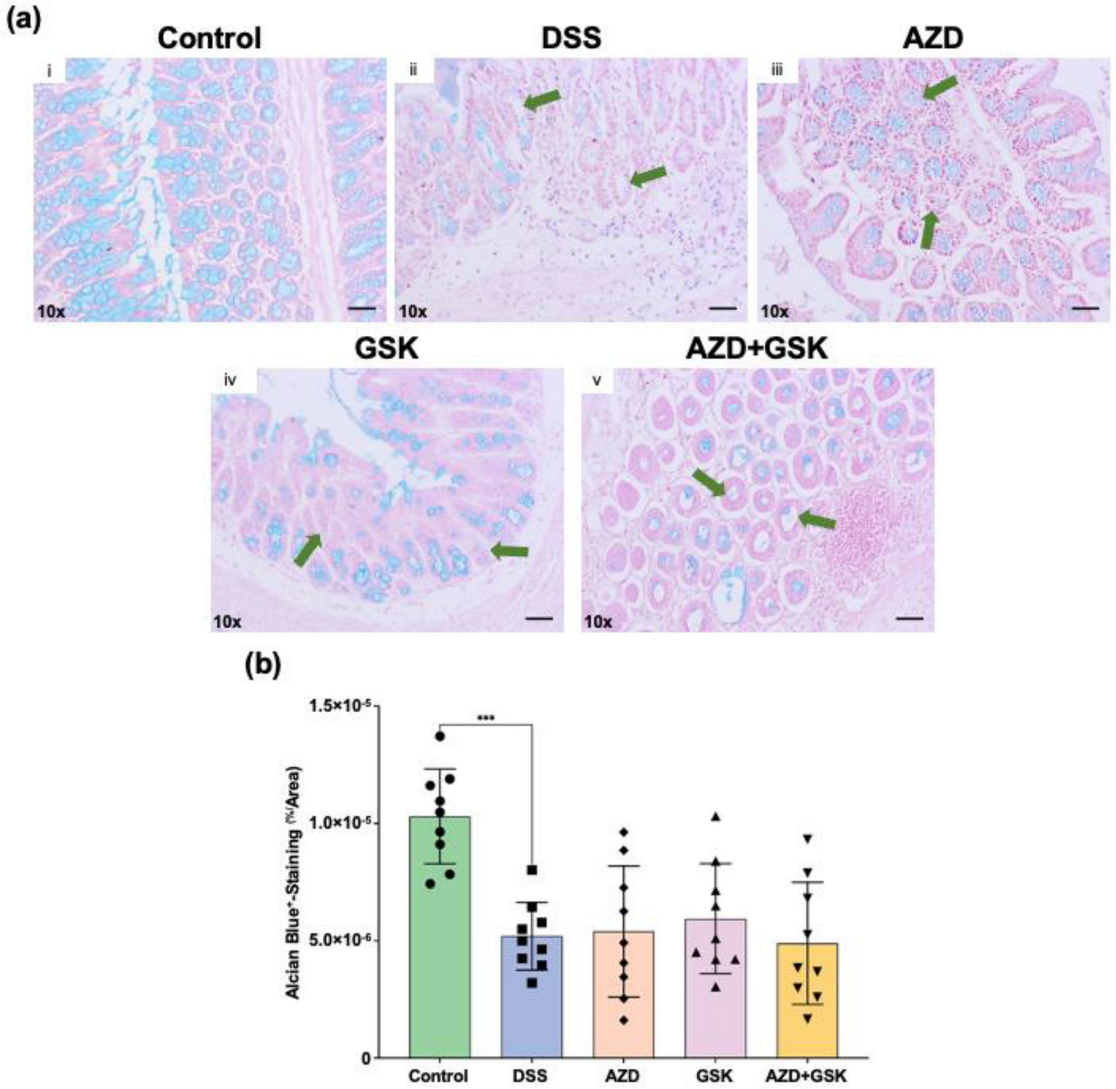
Alcian blue and Safranin O staining for goblet cells and mucin in mouse colons. (**a**) Representative colon images from different experimental groups were captured from Axio Lab.A1 light microscope with a Axiocam 105 Color camera at 20x magnification. (i–v) The area of goblet cell death and mucin loss was highlighted with green arrows. Scale bar = 50 µm. (**b**) Quantification of positive staining for alcian blue is expressed as % of positive stain vs. colon area. Graphical values represent mean ± SD with *n* = 9 mice per group. Normalcy of the collect data was analysed using Shapiro–Wilk test, group difference was analysed by one way ANOVA with Tukey’s multiple comparison as a post hoc test. *P≤0.05, **P≤0.01, ***P≤0.001 and ****P≤0.0001.

We next utilised Alcian blue and Safranin-O stains to investigate the impact of different treatments on mucus-secreting cells from isolated colons. Goblet cells synthesise and secrete mucin to form a protective colonic mucus layer [39]. Along with the extensive inflammatory damage observed in the colons from the DSS group, weak staining of Alcian blue suggested a loss of goblet cell mucin secretion in response to the DSS insults (green arrows in Figure 3a(i-v)). This outcome is further supported by the quantitative analysis of Alcian blue^+^ staining, where significant reduction of Alcian blue was observed in colons taken from the same DSS-insulted mice compared with the control (*P*=0.0003, Figure 3b). In mice treated with pharmacological inhibitors (either separately or in combination), the loss of mucin was persistent and was not recovered to control levels. Percentage (%) of alcian blue^+^ staining from all three treatment groups was not significantly different compared with the DSS group (*P*>0.05), which suggests that MPO and/or PAD4 inhibition did not prevent goblet cell loss and neither mitigated mucin loss nor improved the mucus physical protective layer.

### MPO inhibition potentially facilitates mast cell migration and activation, whilst PAD4 inhibition dampened such mast cell response

**Figure 4:**
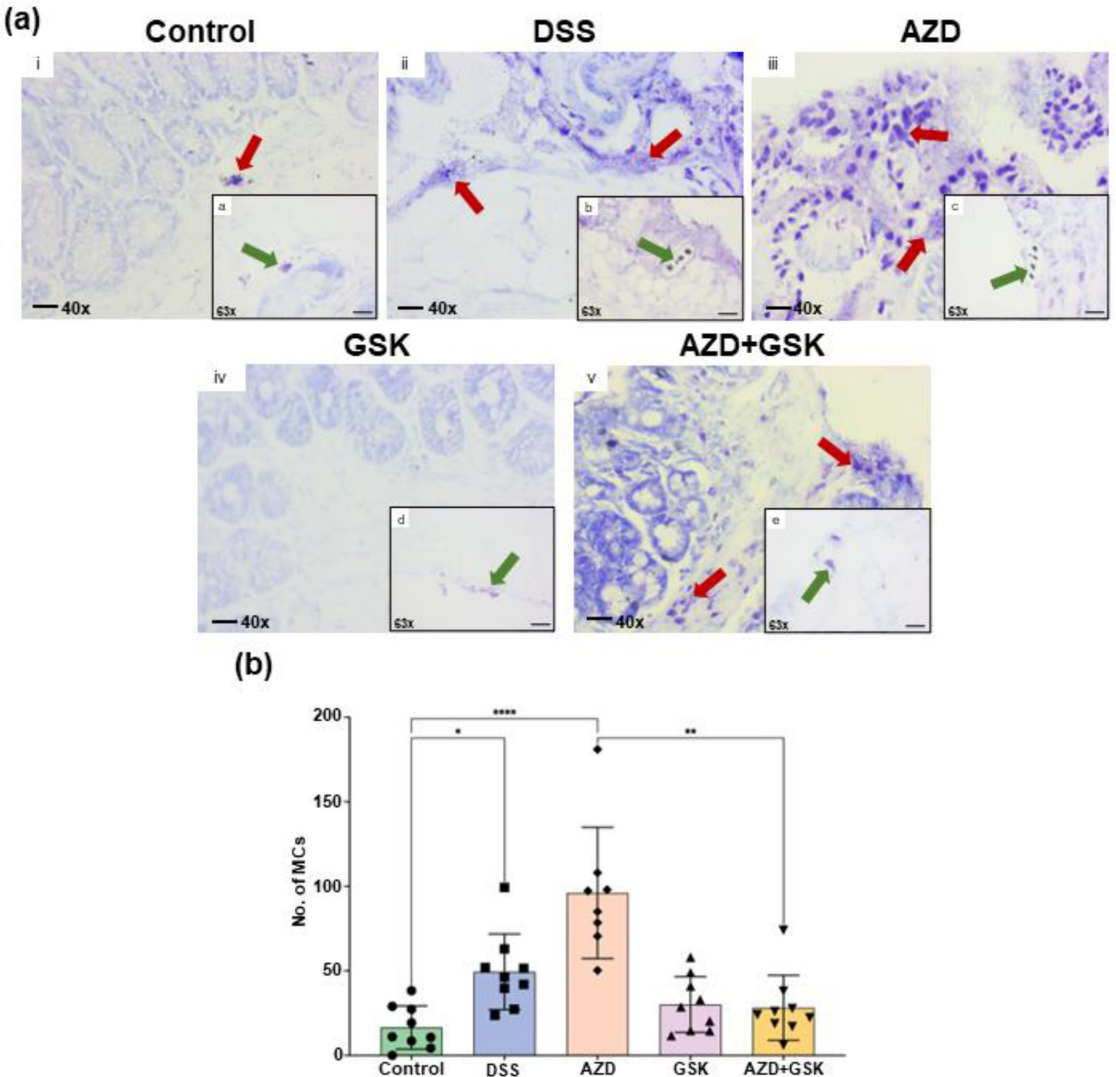
Toluidine Blue staining for mast cells (MC) in colon tissues. (**a**) Representative colon images from different experimental groups. Images were captured from Axio Lab.A1 light microscope with a Axiocam 105 Color camera at 40x and 63x magnifications as indicated on the figure. Scale bar = 20 µm. (**i**) Red arrow showed a single MC in the connective tissue of submucosa and (**i.a**) MC granule was highlighted by the green arrow. (**ii**) Red arrows indicated extensive degranulation of MC in the submucosa and lamina propria layers, (**iii**) but MCs were not degranulated in the AZD group (**ii.b and iii.c**) green arrows displayed the presence of MCs in blood vessels. (**iv**) Absence of submucosal MC was observed in GSK484-treated mouse colons and (**iv.d**) presence of degranulated MC in the muscularis externa layer (green arrow). (**v**) Red arrows showed submucosal connective tissue MC and (**v.e**) presence of an inactive MC was indicated by green arrow. (**b**) Quantification of positive staining for Toluidine blue throughout the entire section. Graphical values represent mean ± SD with *n* = 9 mice per group. Normalcy of the collect data was analysed using Shapiro–Wilk test, group difference was analysed by one way ANOVA with Tukey’s multiple comparison as a post hoc test. *P ≤ 0.05, **P≤0.01, ***P≤0.001 and ****P≤0.0001.

Infiltrating MCs are characteristic of the inflammatory response of IBD and experimental colitis [10,14]. Here, Toluidine blue staining of mouse colon tissues was used to identify MCs. In the control group, few MCs were detected in the connective tissue with little evidence of MC degranulation (Figure 4a(i), respectively). Colons from mice exposed to DSS insult were characterised by substantial MC degranulation in the lamina propria, submucosa and muscularis externa layers (Figure 4a(ii)). Additionally, MCs were identified in blood vessels and in colon adventitia layers (Figure 4a(ii) and b), consistent with MC infiltration and subsequent activation in the colon tissue. Compared with the control, a significant increase in MC number was identified in the colons of mice allocated to the DSS group (*P*=0.0278, Figure 4b). Similar to the DSS group, a pronounced MC response was also observed in mice from the AZD group, where MC count from mice that received the MPO inhibitor was significantly higher than control (*P*<0.0001 Figure 4b). However, most of the MCs were not activated (as judged by an absence of non-degranulated) (Figure 4a(iii) and c). As shown in Figure 4a(iv,v), a notable decrease in MC density was determined when mice were treated with PAD4 or AZD + PAD4 inhibitors. However, these differences were not statistically significant (*P*>0.05, Figure 4b). Together, these results showed the potential involvement of MC in the inflamed colon that paralleled neutrophil recruitment, thereby implicating the possibility of immune-crosstalk with the neutrophil inflammatory pathway.

### PAD4 inhibition diminishes the density of NETs in mucosal crypts evaluated by triple-labelled immunofluorescence

**Figure 5:**
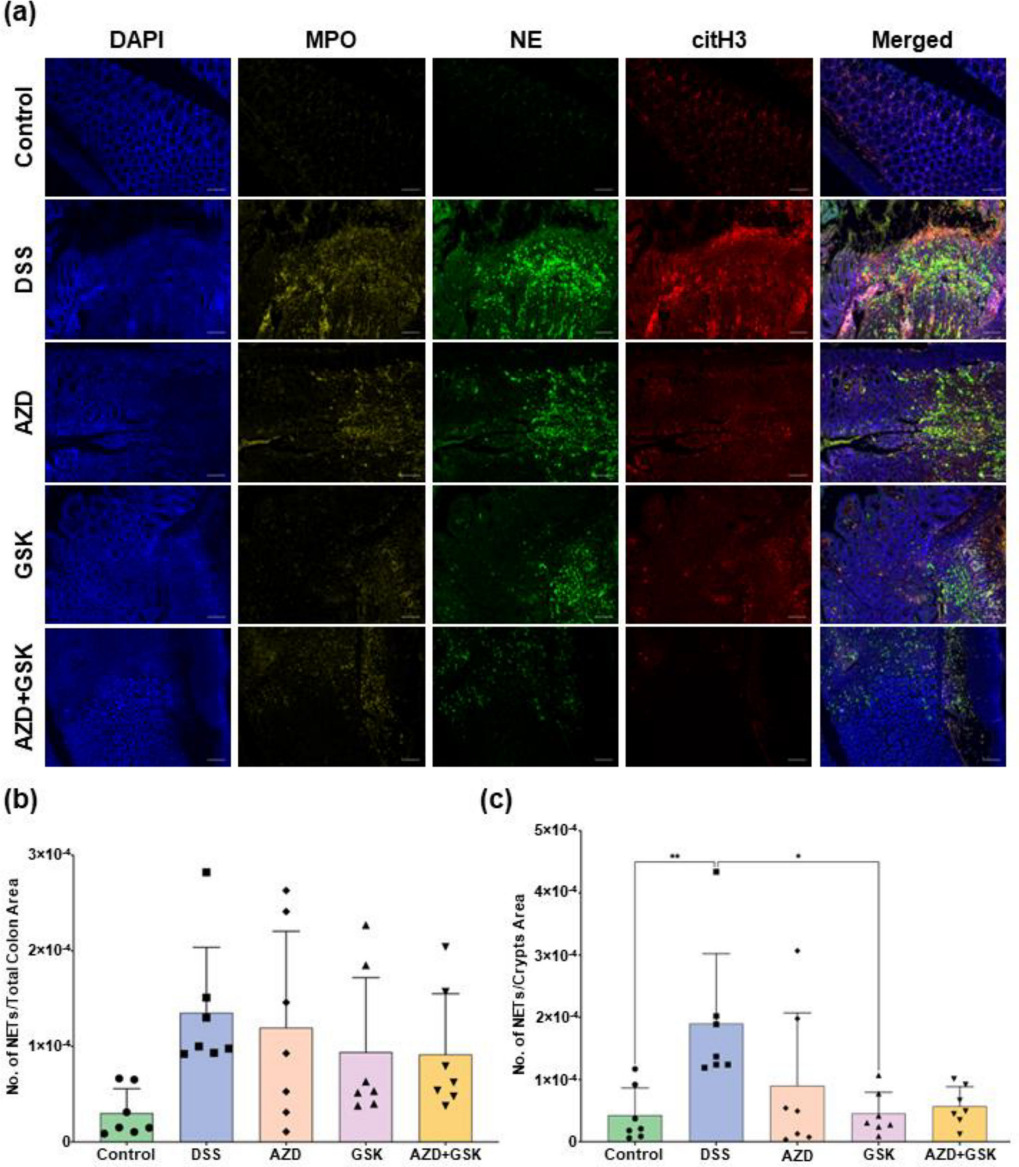
The effect of pharmacological inhibition of MPO and/or PAD4 on the number of NETs in DSS-insulted colons. (**a**) Representative images of triple-plex immunofluorescence images of colons from different experimental groups were captured from Axio Scope.A1 fluorescence microscope with a AxioCam-ICm1 camera at 10x magnification. Scale bar = 100 µm. (**b**) Number of NETs per total colon area. (**c**) Number of NETs per cryptic area in the colon. Statistical outliers were identified and removed using the ROUT method (Q = 1%) and data normality was tested using the Shapiro–Wilk test. Graphical values represent mean ± SD with *n* = 7 mice per group after outlier removal. Kruskal–Wallis test with Dunn’s multiple comparison as a post hoc were performed for non-parametric data. *P≤0.05, **P≤0.01, ***P≤0.001 and ****P≤0.0001. DSS, dextran sodium sulphate; MPO, myeloperoxidase; NETs, neutrophil extracellular traps; PAD4, peptidyl arginine deaminase IV.

To further investigate the relationship between MC and neutrophils in DSS-induced colitis in the presence of MPO and/or PAD4 inhibition, we next examined the extent of colon NETosis. Chromatin decondensation promoted by MPO and NE and concomitant citrullination of the histone proteins by PAD4 are processes essential for NETosis [23,24]. The current study utilised multiplex immunofluorescence (IF) labelling of these three key markers: MPO, NE and citH3 to confirm spatial co-localisation of these proteins in the extracellular domain (Supplementary Figure S4) and to visualise/quantify NETs in colon tissues. As shown in Figure 5a, a noticeable increase in the colon expression of MPO and NE was detected in mice from the DSS-insult group, whereas little expression of these two immune^+^ biomarkers was detected in the healthy controls. Importantly, pharmacological inhibition of MPO and/or PAD4 enzymes resulted in decreased MPO and NE accumulation in the DSS-injured colon. Also, the level of histone citrullination, detected as citH3^+^ immune signal, was relatively higher in the same colon tissue, whilst colons from the healthy controls, AZD, GSK and AZD+GSK treatment groups all displayed similar levels of immune^+^ fluorescence. However, semiquantitative analysis of the IF images showed that none of these visible changes reached statistical significance (Supplementary Figure S5a-f, *P*>0.05).

The current study also attempted to quantify NETs density in colon tissues by examining the degree of overlapping staining of all three immune markers. Importantly, when investigating NETs density specifically in the mucosal (epithelial) region of the colon, it was noted that NETosis increased significantly in DSS-treated colons (*P*=0.0092), whereas pharmacological inhibition of PAD4 with GSK484 significantly lowered NETs density in the same colon region (*P*=0.0294, Figure 5b and c). The administration of AZD3241 (alone) and AZD3241+GSK484 (combined) also lowered mucosal NETs formation, albeit this did not reach statistical significance (*P*>0.05). Thus, inhibition of PAD4 with GSK484 showed different regional effects, and reduction in NETs was limited to the colon mucosa.

### MPO and/or PAD4 inhibition marginally alters antioxidant signalling proteins without affecting colonic lipid peroxidation

**Figure 6:**
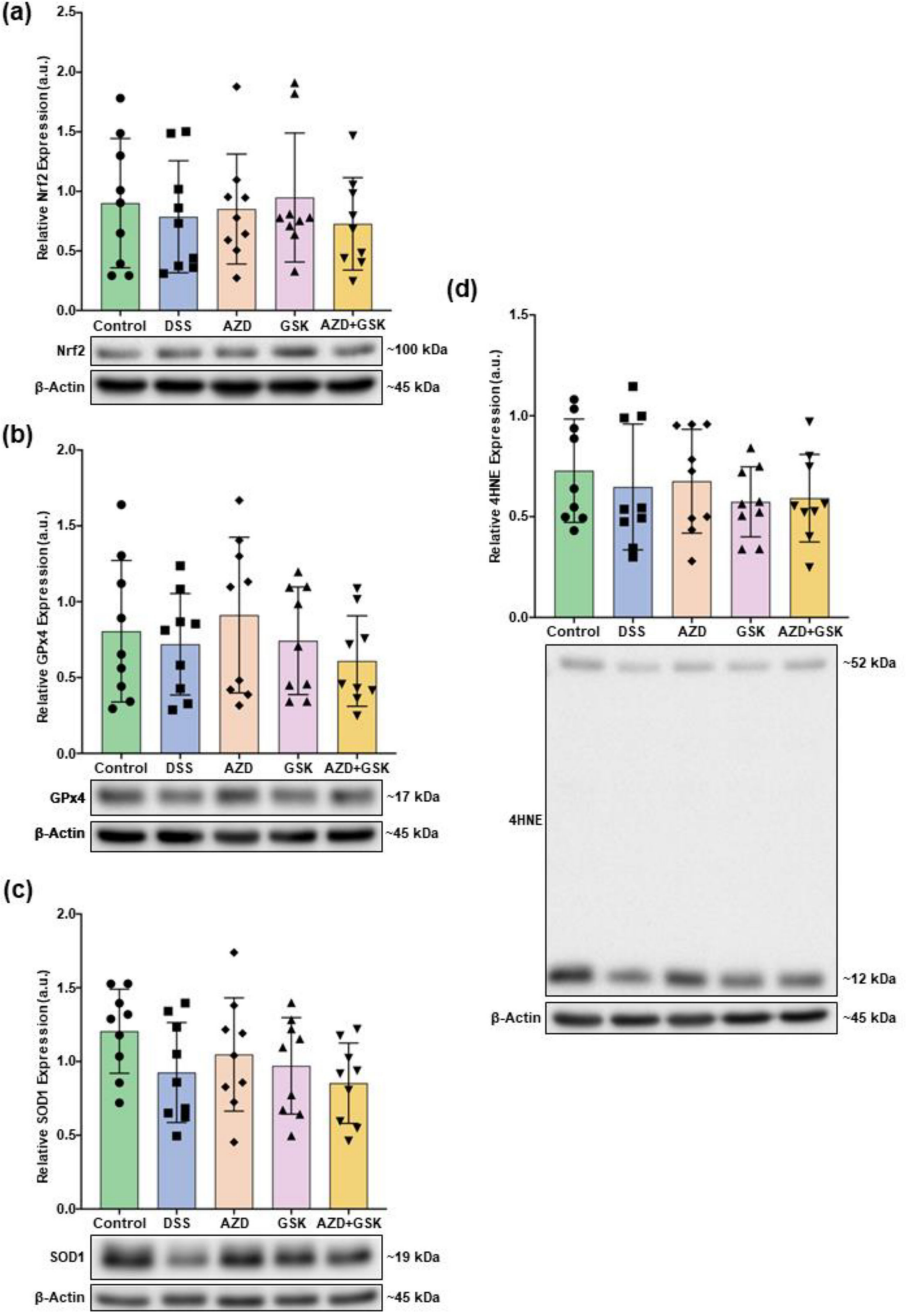
The effect of pharmacological inhibition of MPO and/or PAD4 on antioxidant signalling protein expressions and lipid peroxidation in colon tissue. Representative western blots bands with densiometric quantification graphs of (**a**) Nrf2, Nuclear factor erythroid 2-related factor 2. (**b**) GPx4, glutathione peroxidase 4; (**c**) SOD1, superoxide dismutase-1; and (**d**) 4HNE, 4-hydroxynonenal. Relative expression (densiometric value) was quantified as an intensity ratio of protein of interest/β-actin. Graphical values represent mean ± SD with *n* = 9 mice per group. Normalcy of the collect data was analysed using Shapiro–Wilk test, group difference was analysed by one way ANOVA with Tukey’s multiple comparison for parametric data and Kruskal–Wallis test with Dunn’s multiple comparison test was used for non-parametric data distributions. MPO, myeloperoxidase; PAD4, peptidyl arginine deaminase IV; SOD, superoxide dismutase.

Previously published studies have reported redox state dysregulation in both IBD patients and animal models of experimental colitis [40-42]. Next, we investigated the effect of pharmacological inhibition of MPO and PAD4 on transcription factors and enzymes that are involved in the antioxidant signalling pathway. As shown in Figure 6a, DSS supplementation in drinking water did not change the colonic expression of Nrf2, and administration of PAD4 inhibitor showed a non-significant trend to increase Nrf2 expression compared with the control group (*P*>0.05). As indicated in Figure 6b, protein expression of GPx4, a downstream antioxidant enzyme regulated by Nrf-2 transcriptional activation [43], trended to increase in the AZD group, although this was not significantly different to all groups (*P*>0.05). SOD1 is an antioxidant enzyme with profuse expression in the gut, in which it modulates intestinal redox homeostasis under physiological conditions [44]. Despite not reaching statistical significance, Figure 6c showed that DSS insults resulted in a trend to decreased expression of colonic SOD1 (*P*>0.05).

By contrast, the inhibition of MPO or PAD4 mitigated this trend of reduction in SOD1 expression in the colon. However, this difference was not statistically significant when compared with mice treated with DSS alone (*P*>0.05). In addition to the antioxidant signalling proteins, we investigated the effect of MPO and/or PAD4 inhibition on lipid peroxidation by using 4HNE as a marker. Group-wise comparison demonstrated that the difference across all experimental groups was not statistically significant (*P*>0.05, Figure 6d). Overall, western blot studies on antioxidant proteins and oxidative stress markers suggest that MPO and/or PAD4 inhibition had minimal influence on the redox protein expression in the DSS-insulted colons. Contrary with previous studies that reported altered redox protein expressions in the model of DSS-induced experimental colitis, the utilisation of total colon tissue (which encompasses inflamed as well as non-inflamed areas) as well as acute induction of the disease may account for this negligible change.

### PAD4 inhibition reduced total SOD activity in colons but had no effect on catalase activity

**Figure 7:**
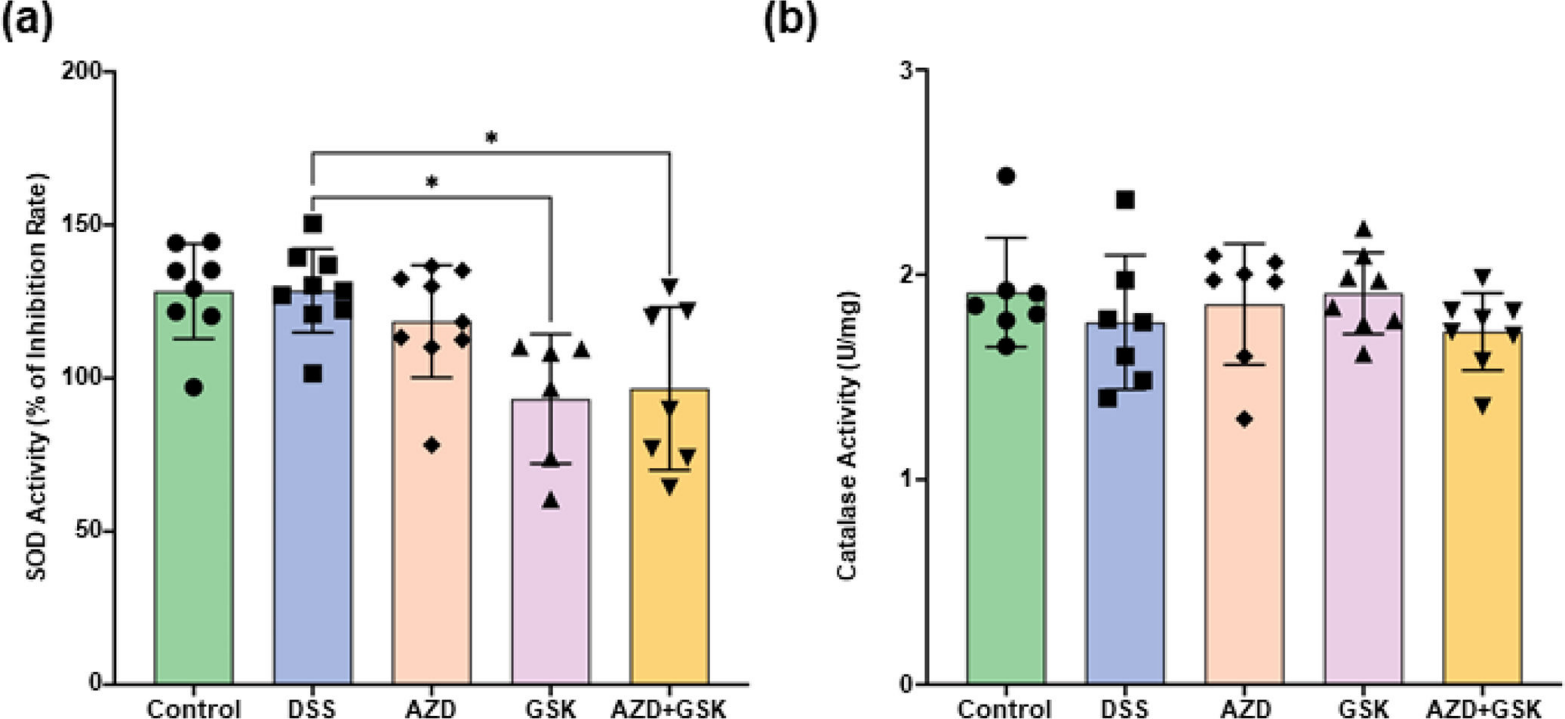
The effect of MPO and/or PAD4 inhibition on antioxidant enzyme activities. (**a**) Total superoxide dismutase (SOD) activity. (**b**) Total catalase activity. Graphical values represent mean ± SD with *n* = 6–9 mice per group after standard curve interpolation. Normalcy of the collect data was analysed using Shapiro–Wilk test, group difference was analysed by one way ANOVA with Tukey’s multiple comparison as a post hoc test. *P≤0.05, **P≤0.01, ***P≤0.001 and ****P≤0.0001. MPO, myeloperoxidase; PAD4, peptidyl arginine deaminase IV.

To further explore colonic SOD1 expression in the DSS-supplemented mice (refer to Figure 6c), total SOD activity assay was determined in the same colon tissue. As shown in Figure 7a, total SOD activity was maintained in the colon when compared with the controls, suggesting a potential up-regulation of SOD enzymatic activities as a compensatory mechanism for the reduction in its expression. However, in the mice that received PAD4 inhibitor or MPO and PAD4 inhibitors simultaneously, a significantly lower SOD activity was observed (*P*=0.0104 and *P*=0.0168 respectively). A similar trend to lower total SOD activity was observed in mice supplemented with the MPO inhibitor, albeit this did not reach statistical significance (*P*>0.05). These results suggest that NET inhibition by PAD4 (refer to Figure 5) could potentially diminish total SOD activities in the colitis colons. As SOD catalyses the dismutation of superoxide radicals into oxygen and hydrogen peroxide [45], total catalase activity was evaluated to understand the colon capacity to neutralise ROS in response to NETs inhibition by PAD4. Overall, no difference in catalase activity was observed across all experimental groups (Figure 7b).

### MPO and/or PAD4 inhibition has minimal effect on IL-1β level in DSS-stimulated colons

**Figure 8:**
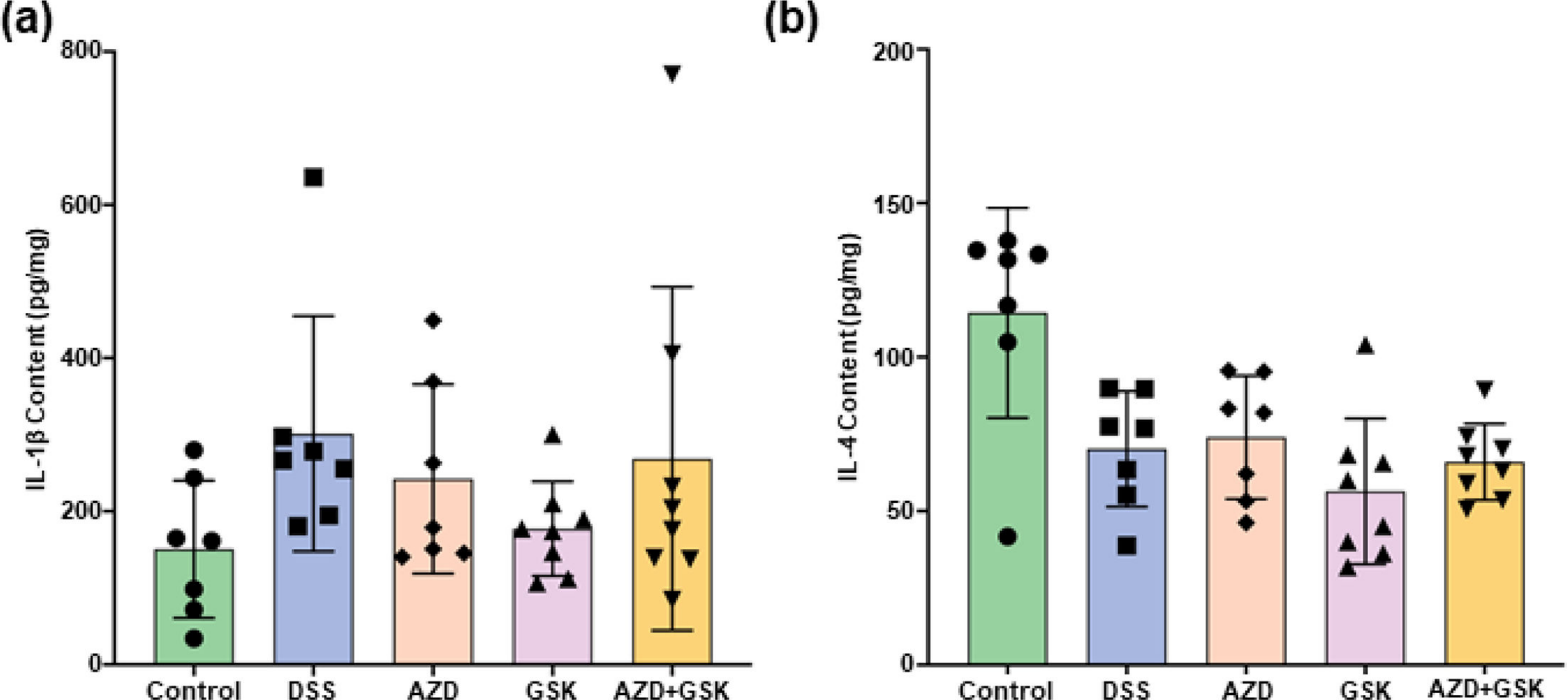
The effect of MPO and/or PAD4 inhibition on inflammatory markers. (**a**) Interleukin (IL)-1β. (**b**) IL-4. Graphical values represent mean ± SD with *n* = 7–8 mice per group after standard curve interpolation. Normalcy of the collect data was analysed using Shapiro–Wilk test, group difference was analysed by one way ANOVA with Tukey’s multiple comparison for parametric data and Kruskal–Wallis test with Dunn’s multiple comparison test was used for non-parametric data. MPO, myeloperoxidase; PAD4, peptidyl arginine deaminase IV.

Finally, we investigated the effect of MPO and/or PAD4 inhibition on the change in immunological profiles by examining the balance of pro-inflammatory (IL-1β) and anti-inflammatory (IL-4 and IL-10) cytokines in isolated colon. A trend to increased colon IL-1β was observed in the mice that were challenged with DSS. However, this was not statistically significant when compared with the healthy controls (*P*>0.05). Contrastingly, treatment with MPO and/or PAD4 inhibitors appeared to diminish the marginal increase in IL-1β level in the colon (*P*>0.05. Figure 8a). DSS supplementation has shown to drastically dampen the anti-inflammatory profiles in the colon tissue. Overall, IL-10 was significantly downregulated (*P*<0.0001, Supplementary Figure S6), whilst a substantial decrease in colon IL-4 level was also observed, albeit not statistically significant when compared with the DSS group (*P*>0.05, Figure 8b). No difference in colon IL-4 and IL-10 levels was detected between the DSS and mice co-supplemented with MPO and/or PAD4 inhibitors (*P*>0.05), suggesting that AZD3241 and GSK484 have no effect on IL-4 and IL-10 molecular pathways.

## Discussion

Whilst current evidence supports the notion that excessive neutrophil infiltration and dysregulated immune responses are linked to IBD disease pathogenesis, the precise mechanism remains unclear. In the context of UC, the formation of NETs can be considered a process that maintains the DNA-damage activity by trapping MPO released from neutrophils within the extracellular matrix, effectively prolonging MPO activity and sustaining inflammation in the colon [26]. Accordingly, elevated NET formation and parallel enhancement of inflammation have been reported in the colon mucosa of patients with UC [29]. Therefore, the inhibition of extracellular MPO activity and/or decreased NET formation can potentially represent a therapeutic approach to improve tissue damage observed in the pathogenesis of UC. This current study was the first to highlight the spatial co-localisation of 3 essential NETosis enzymes: MPO, NE and citH3 in the colon tissue using multi-plex IF imaging, further reinforcing the involvement of NETs during the pathogenesis of DSS-induced experimental colitis. As anticipated, DSS insult elevated NET density in colon tissue, yielding a significant increase in the colonic crypts, indicating that NET formation occurs primarily in the mucosa layer during acute inflammation in UC. Similarly, *Citrobacter rodentium –* a murine pathogen that mimics *Escherichia coli* infection – has also been shown to elicit NET formation in specific experimental mouse strains. For instance, *Citrobacter rodentium*-infected C3H mice display similarities clinical and histological features similar to those observed in UC. A study by Sanchez-Garrido et al. demonstrated that a greater number of neutrophils was evident in the lumen of C57 mice, whereas an extensive accumulation of neutrophils and a marked elevation of NETs and citrullinated histone H3 were observed in *C. rodentium*-infected C3H mice, specifically trapped within the colon mucosa and submucosa [46]. These findings are consistent with our results, where DSS-induced chemical colitis also led to acute inflammation, elevated faecal CP and triggered NET formation in the colonic mucosa. We also showed that administration of GSK484 (inhibiting PAD4) at 4 mg/kg administered four times over 9 days (total drug provided to each mouse ~400 μg) significantly reduced mucosal NETs. Meanwhile, the treatment with either AZD (MPO inhibitor) or GSK484+AZD trended to decrease mucosal NET formation, although this was not significantly different to mice stimulated with DSS alone. Despite GSK484 reducing mucosal NET density, the extent of UC-like experimental colitis remained unchanged as judged by several measures of colon inflammation, suggesting a local effect without affecting disease pathogenesis.

AZD3241 is a synthetic MPO inhibitor developed by AstraZeneca, in which it completely blocks MPO-mediated oxidation at 2 µM and elicits minimal effects on the oxidation activities of other peroxidases such as thyroid peroxidases and lactoperoxidases [47]. Despite a compensated dose being administered, the colon protective activity of AZD3241 (inhibiting extracellular MPO) was not recapitulated in the current study, evident in the lack of improvement in experimental colitis symptoms and persistent intestinal inflammation after the mice were co-supplemented with DSS and AZD3241. This may be attributed to the presence of low-molecular weight contaminants in the newly acquired compound, interfering with previously reported AZD3241 bioactivity [33]. Despite adjusting the dosage of AZD3241 to closely match dosing used previously [33], a potential limitation is the low purity of the compound used in this study, which may restrict the anti-inflammatory action of this inhibitor or elicit unwanted pro-inflammatory activity in colon tissues from low-molecular weight contaminants. The effect of AZD3241 on NET formation has been studied in the context of experimental UC. Interestingly, a markedly lower number of NETs was observed in the mice that received AZD3241 treatment, suggesting that the MPO inhibitor reduces NETosis via a pathway that is PAD4-independent, possibly via reduced oxidative stress in the colon mucosa. Indeed, AZD3241 has shown to reduce oxidative stress in the brain of Parkinsonian patients [48]. This is further supported by the critical roles of ROS in NETosis, where MPO-derived ROS are documented to stimulate neutrophil elastase translocation to the nucleus via the MEK-extracellular-signal-regulated kinase (ERK) signalling pathway [49,50]. Nevertheless, additional research is required to fully understand the effect of inhibiting MPO activity on NETs formation in IBD.

On the other hand, GSK484 is a selective inhibitor against the PAD4 enzyme, and it has been shown to have negligible off-target activities against a panel of 50 unrelated proteins [51]. Previous reports have demonstrated that the administration of PAD4 inhibitors like GSK484 at the same dose of our study (4 mg/kg) but delivered daily for one week suppressed NETosis in mice with cancer-associated kidney injury [52]. Meanwhile, an intraperitoneal injection of GSK484 at a higher concentration (10 mg/kg body weight) in a murine model of myocardial infarction resulted in profound inhibition of NETosis and significantly improved clinical parameters with no adverse side effects [53]. These successful experimental interventions may be due to GSK484 being tested under different dosing regimens (daily administration vs. every second day in the current study), suggesting that a greater extent of PAD4/NETs inhibition is required to achieve a threshold level of NETs inhibition and consequently a therapeutic effect. Additionally, the pharmacokinetics of GSK484 display a low-moderate clearance rate yielding a half-life (T1/2 h) of 3.8 ± 1.5 h and blood clearance (Clb) over 19 ± 3 ml/min/kg in mice [51,54], showing that daily administration could be beneficial to achieve optimal pharmacological activity. Nevertheless, the dose tested here was able to limit NETosis in the colon mucosa, although this focal inhibition failed to ameliorate disease progression.

It is notable that the therapeutic potential of NETs inhibition by limiting PAD4 activities should be examined with caution, as the available literature reports conflicting results on the role of NETosis in IBD development. In the study by Dragoni et al., NETs were identified to be a potential pathological stimulus for fibroblast activation, and depletion of PAD4 in neutrophils reduced NET formation and limited fibroblast activation, implying a stimulatory role of PAD4 in mediating fibrogenesis in IBD [55]. In contrast, Leppkes et al. reported that PAD4-deficient mice were associated with exacerbated DSS-induced colitis and more severe rectal bleeding, suggesting a vital role for PAD4 and NETs in minimising immuno-thrombosis and rectal bleeding [56]. These outcomes conflict with the data reporting PAD4 inhibition with a pan-inhibitor can ameliorate experimental colitis [34, 35]. Together, these findings suggest that the therapeutic potential of PAD4-dependent NETs inhibition in IBD remains unclear and warrants further investigation. Certainly, more research is required to unambiguously demonstrate that inhibiting NETosis is an appropriate therapy for treating IBD and to establish the optimal dosage of PAD4-specific inhibitors such as GSK484 without generating associated risks like opportunistic infection.

The role of MC is not fully clear in the pathogenesis of IBD. Inflammatory and oxidative stimuli can act in concert to recruit MC and stimulate neutrophils to perpetuate a continuous cycle of inflammatory immune cell infiltration and driving the chronicity of the disease [57], particularly in the colon mucosa. Interestingly, production of the potent MPO-oxidant HOCl chlorinates the primary amino group of histamine derived from MC to form chloramine-histamines [58], thereby potentially limiting HOCl-mediated oxidation [59] in the colon mucosa. In this study, mice simulated with DSS insult showed concomitant increases in NETs and MC in the colon. This result highlights that MC migration/degranulation occurs in parallel with active recruitment of neutrophils during the pathogenesis of DSS-mediated colon inflammation, suggesting that MC mediators and downstream histamine-modification by HOCl could be a potential protective compensatory mechanism to prevent indiscriminate HOCl-mediated colon damage. This notion is supported by a study in an IL-10-deficient mouse model of IBD, where colonic MCs were found to enhance intestinal epithelial barrier function and protect the colon mucosa [60]. Notably, accompanying a reduction in NETosis in the colon mucosa, treatment with GSK484 simultaneously and significantly reduced the number and degranulation status of MC in the same colon region, with levels diminished to be similar to controls. This outcome may explain the lack of improvement in symptoms and biomarkers of experimental colitis in the presence of the PAD4 inhibitor. Therefore, this current study shows for the first time another potential pathway in the non-allergic regulatory role of MC, suggesting that GSK484 failed to resolve intestinal inflammation and colitis symptoms, likely due to the inhibition of MC activation. This is further supported by previous studies that have shown the immunomodulatory and protective role of MC in attenuating injury and inflammation [61,62].

Furthermore, the close relationship between MC and neutrophils has been recently reported in chronic allergic inflammation. Interestingly, MC degranulation can reroute neutrophil migration, leading them to invade MC and initiating a process where neutrophils become trapped by MC and form ‘cell-in-cell’ structures, where neutrophils can remain viable up to 48 h post encapsulation inside the MC. This newly described phenomenon called MC intracellular trap (MIT) by Mihlan et al. [63], which suggests that MC can prolong neutrophil survival in tissues and may play a relevant role in the inflammatory process that involves the innate immune response; although it remains to be determined whether the PAD4 inhibitor (GSK) not only reduced NET formation but also inhibited MC migration and activation, which in turn affects MIT structures. In contrast, the study by Kurashima et al. demonstrated that MC activation promotes DSS-induced experimental colitis via P2X7 receptors [64], whilst Okayama et al. showed that neutrophils promote intestinal inflammation via MC infiltration and activation in a rat model of indomethacin-induced enteritis [65]. However, these outcomes were observed in a period of 24 hours after inflammation induction. The current study observed a significant increase in MC when mice were treated with AZD3241, suggesting that neutrophils may potentially activate MC through other pro-inflammatory cytokines such as IL-18 [66], which has been previously demonstrated to be critical for MC activation and degranulation in inflamed tissues *via* IL-18R [17]. Thus, further work is warranted to elucidate the potential interplay between non-allergic function of MC and neutrophils to achieve a comprehensive understanding of the interplay between these cell types in the setting of IBD.

Nrf2 governs the transcription of numerous genes involved in the antioxidant defence system to maintain physiological redox balance. For instance, Nrf2 promotes the expression of thioredoxin and thioredoxin reductase to facilitate the removal of oxidised thiols and peroxides [67,68] whilst regulating the expressions of glutamate cysteine ligase and GPx to allow glutathione production and ROS detoxification [69,70]. However, this redox balance was disrupted in UC, where impaired activities of SOD, GPx and CAT in UC all contribute to chronic inflammation in the gut [71]. Additionally, oxidative stress is also closely interrelated with NETs formation, where NADPH oxidase-generated ROS is required for the nuclear translocation of NE during the process of NETosis [24]. Interestingly, this current study showed that the administration of AZD3241 and/or GSK484 lowered the total SOD activities in the homogenised colon tissues, suggesting that reduced colon mucosa NETs formation is associated with lowered ROS detoxifying power in the gut. However, a major limitation in the current study is the lack of examination on ROS content, where the levels of H_2_O_2_ and MPO-derived HOCl were not directly evaluated. As a result, the link between intestinal NETs reduction and diminished SOD activities in the context of DSS-induced experimental colitis requires further investigations where the potential synergistic effects of quenching ROS as well as administration of NETosis-associated enzyme inhibitors should also be explored.

To conclude, the current study highlighted the involvement of NETs in the DSS-induced model of experimental colitis via multi-plex IF imaging, whilst the administration of AZD3241 (inhibiting MPO) and GSK484 (inhibiting PAD4) both reduced NET mucosa formation and reduced MC migration and activation, which are closely related to the pathophysiology of the neutrophil immune response. Overall, the off-target effects of these drugs yielding changes in MC responses may explain why inhibiting PAD4 activity and reducing mucosal NETosis failed to improve clinical symptoms nor reduce intestinal inflammation.

## Methods

### Animals

All experimental procedures involving mice were conducted within the Laboratory Animal Service (LAS) facility at the University of Sydney and followed the approved protocol by the University of Sydney Animal Ethics Committee (Approval #2019/1496). Male C57BL/6 mice (six weeks of age) were purchased from Animal Resource Centre (Perth, Australia) and housed in environmentally enriched ventilated cages at the LAS facility located at the Charles Perkin Centre (CPC) at the University of Sydney, Australia, under a 12-h light–dark cycle at 22°C with standard chow diet and tap water provided *ad libitum*. All mice were acclimated for seven days prior to the start of experiments, and each mouse was tail marked for individual identification.

### Drug administration

Molecular grade DSS (molecular weight range: 36–50 kDa) was purchased from MP Biomedicals (CAS# 9011–18-1). Acute experimental colitis was induced with 2% w/v DSS in the drinking water as previously described by our group [33]. Water consumption was monitored daily, and fresh DSS-water mixture was replaced every 3 days with the same dosage maintained throughout the duration of the study. The synthetic MPO inhibitor, AZD3241 (also known as Verdiperstat), was purchased from MedChemExpress (Cat# HY-17646), whilst PAD4 inhibitor, GSK484, was acquired from a commercial source (Abcam, Sydney Australia, Cat# ab223598; purity > 98%).

The doses and administration routes for MPO and PAD4 inhibitors were selected based on previously published studies [33,72], taking into consideration the need to avoid toxic effects and high doses that could lead to consequences of infections, since MPO activity/NETosis is associated with the bactericidal action of MPO. A stock solution of GSK484 (25 mg/ml) was prepared in 100% ethanol and stored at −30℃. Where required, this PAD inhibitor was diluted with sterile saline using an insulin syringe (Thermo) and administered via *i.p*. injection to achieve a final dose of 4 mg/kg body weight. Additionally, mice were trained to accept approximately 0.1 g of peanut butter (PB) via oral intake during their acclimation period to avoid invasive oral gavage of AZD3241 and hence reduce animal handling-associated stress.

### Electrospray mass spectrometry analysis of AZD3241

Recently, the colon protective activity of the MPO inhibitor AZD3241 (obtained as a gift from Pharmaxis Ltd, Frenchs Forrest, Sydney) was demonstrated in an experimental colitis [33]. Due to a change in supplier, and to validate the purity of the MPO inhibitor supplied by MedChemExpress, electrospray ionisation mass spectrometry was conducted on both original and newly sourced AZD3241 using a Q Exactive HF-X Orbitrap System (Thermo Scientific). Both inhibitors were analysed at 5 µg/mL in 50% v/v methanol, and the Q Exactive HF-X Tune Software (Thermo Scientific) was used to operate the orbitrap system in positive ion mode with a HILIC column. Data accumulation parameters were as follows: scan range: 50.0–750.0 m/z, mass resolution 120,000, spray voltage: 4 kV, capillary temperature: 320°C and Funnel RF level: 50. Both synthetic drugs were diluted 1:200 v/v in H_2_O: MeOH = 1:1 v/v (final concentration ~5 μg/mL). Analysis was performed in triplicate and peak intensities were averaged and compared. The amount of MedChemExpress-supplied AZD3241 administered to the mice was then normalised through relative comparison to the authentic AZD3241 supplied by Pharmaxis to achieve an equivalent dose.

### Experimental design

**Figure 9:**
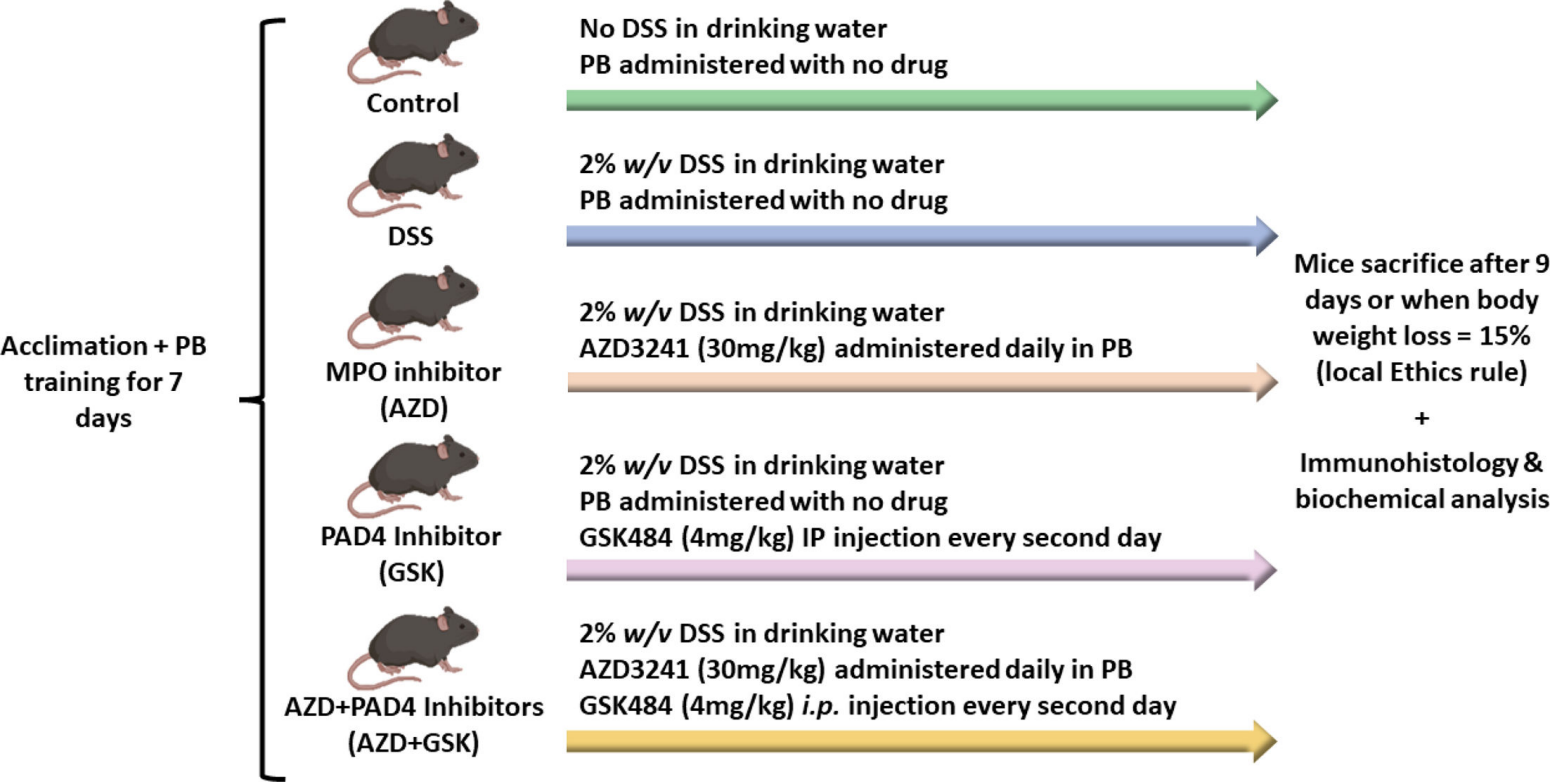
Overview of the experimental timeline and group allocations. (i) Control group; (ii) DDS group, dextran sodium sulphate (DSS) at 2% w/v was administered to induce colitis; (iii) AZD group, myeloperoxidase (MPO) inhibitor AZD3241 at 30 mg/kg was provided to mice daily in peanut butter (PB) through the experiment; (iv) GSK group, peptidyl-arginine deiminase IV (PAD4) inhibitor GSK484 at 4 m/kg was injected via i.p. every second day. (v) AZD+GSK inhibitors group, both treatments were administered following the conditions mentioned above.

The experimental design has been outlined schematically in Figure 9. At seven weeks of age, mice were randomly allocated into five groups (*n* = 9 per group) as follows:

1. *Control Group*: 0.1 g of PB daily + standard chow diet and drinking water *ad libitum*.
2. *DSS Group*: 0.1 g of peanut butter daily + standard chow diet and 2% w/v DSS in drinking water *ad libitum*.
3. *AZD3241 Group*: 30 mg/kg body weight in 0.1 g of PB daily + standard chow diet and 2% w/v DSS in drinking water *ad libitum*.
4. *GSK484 Group*: 4 mg/kg injected *i.p*. every second day +0.1 g of PB daily + standard chow diet and 2% w/v DSS in drinking water *ad libitum*.
5. *AZD3241+GSK484 Group*: 4 mg/kg of GSK484 injected *i.p*. every second day+30 mg/kg body weight of AZD3241 in 0.1 g of PB daily + standard chow diet and 2% w/v DSS in drinking water *ad libitum*.

Mice were terminated at the loss of 15% of their body weight measured at day 0 or eight days after DSS induction according to the approved Ethics protocol. At the end of the experiment, blood was collected via cardiac puncture from mice under full anaesthesia (4% v/v inhaled isoflurane). Subsequently, cervical dislocation was performed, and colon tissue and faecal material were harvested.

### Clinical features and disease activity index

Monitoring progression of experimental colitis throughout the duration of the study was achieved by evaluating an individual mouse DAI using criteria previously described [33]. To capture gross disease progression, the average total DAI score for each allocated group was determined at the end of the experiment. The DAI score involved four clinical markers summarised in Table 1 and took into consideration stool appearance, the presence of rectal prolapse, grooming and the percentage of weight loss with scores ranging from 0 to 2. Following organ harvest, the length and colon weight were recorded.

**Table 1:** Disease activity index (DAI) scoring criteria.

Clinical markers	Score	Description
**Stool appearance**	0	Normal
	1	Soft
	2	Watery/Presence of blood
**% of weight loss**	0	< 1%
	1	1–10%
	2	> 10%
**Rectal prolapse**	0	No prolapse
	1	Prolapse present
**Grooming**	0	No hunched posture, bristle fur, or skin lesions
	1	Presence of hunched posture, bristle fur and skin lesions

#### Tissue fixation, embedding and sectioning

Isolated colons were processed initially by overnight fixation in 70% v/v ethanol, followed by embedding in paraffin wax. Next, colons were sectioned at 5 µm thickness with a rotary microtome (Shandon Finesse 325, Thermo), and sections were then mounted onto Superfrost™ Plus Microscope Slides (Fisher Scientific). Mounted slides were dried in an oven (60℃, 2 h). After drying, slides were assigned randomly generated codes to blind the treatment conditions throughout the duration of subsequent staining and image analysis.

### Histopathological studies

Where required, slides were dewaxed and rehydrated in xylene (2 × 10 min) and graded alcohols (2 × 2 min 100% ethanol, 2 × 2 min 95% ethanol and 1 × 2 min 70% ethanol) before commencing staining described below. Histological images were captured by using the Axio Lab.A1 light microscope (ZEISS) with the Axiocam 105 Color Camera (ZEISS). Two imaging fields per section (6 images per slide) were generated at 20× magnifications to conduct histology scoring (H&E and Alcian Blue/ Safranin O staining). Quantitation of MC in Toluidine Blue-stained sections was performed by screening the complete colon section with representative images captured using 40× and 63× objectives by three different experienced researchers (K.X, T.O.C, and J.H) unless specified otherwise.

#### Haematoxylin and eosin staining

Colon histoarchitectural and histopathological changes were assessed using haematoxylin and eosin (H&E)-stained colon sections. Briefly, colon sections (10 µm) were immersed in filtered Harris Haematoxylin solution for 2 min to visualise the cell nuclei, washed thoroughly with tap water, then submerged in Scott’s blue solution for 30 s and another 10 s in acid alcohol. Subsequently, the slides were placed into eosin for 30 s. Next, slides were exposed to 10 s of 95% v/v ethanol and 2 × 10 s of 100% ethanol. After this dehydration step, colon sections were cleared in xylene and mounted with Dibutylphthalate plasticiser xylene (DPX) mounting medium. The criteria for histoarchitecture examination included visualisation of crypt inflammation and loss, degree of neutrophil infiltration and loss of surface epithelium, which were scored manually from 0 to 3, with this scale corresponding to an absence of the criteria to severe damage (refer to Table 2 for scoring summary).

**Table 2:** Haematoxylin and eosin (H&E) histoarchitectural evaluation criteria.

Score Pathologies	0	1	2	3	4
Crypt loss	Intact crypts	Disoriented crypts	Variable crypt diameter	Atrophied crypts	Mucosa devoid of crypts
Neutrophil infiltration	No infiltration	Mucosal/lamina propria infiltration	Mucosal and submucosal infiltration	Moderate cryptitis/infiltration to crypts	Severe cryptitis
Loss of surface epithelium	Intact surface epithelium	Sloughing off epithelial surface	Patchy loss of surface epithelium	Moderate loss of surface epithelium	Severe loss/erosion of surface epithelium

#### Alcian Blue with Safranin O staining

Alcian Blue and Safranin O stains were used to examine the presence of mucin secreted from goblet cells in the colon mucosa. Briefly, slides were immersed in 0.1% w/v Alcian Blue pH 2.5 solution for 30 min before rinsing with distilled water for 5 min. Then, slides were counterstained in 0.1% w/v acetic Safranin O solution for 10 min, followed by another 5 min of distilled water wash to visualise the colon epithelial histoarchitecture. Then, the slides were air-dried completely under a fume cupboard for 4 h before immersing in xylene for 2 × 10 min and coverslipped with DPX medium. The staining intensity of Alcian blue was quantified by two different researchers (K.X and T.O.C) using the ‘Color Threshold’ function in the ImageJ software (v.1.54d, National Institute of Health, U.S.A.).

#### Toluidine Blue staining

Toluidine Blue staining was utilised to determine the presence of resident MC due to their metachromatic properties after staining. The staining procedure was modified from a previously published protocol [13]. Briefly, colon sections were stained with Toluidine Blue working solution (0.5% w/v Toluidine Blue and 1% v/v glacial acetic acid in distilled water) for 90 s. Next, the slides were immersed in visualising solution (5% w/v ammonium molybdate in distilled water) for 5 min to minimise dye removal, rinsed with tap water for 5 min then rapidly dehydrated through graded alcohols (1 × 1 min in 90%, 95% and 100% v/v ethanol with gentle agitation). After 2 × 5 min of clearing with xylene, stained sections were then coverslipped with DPX mounting medium. Since matured MCs often reside in lamina propria, submucosa, smooth muscle or near blood vessels of the gastrointestinal tract [73], the current study focused on the transient MC populations restricted to connective tissue. Furthermore, mucosa and brush border MC staining were excluded due to the non-specific metachromasia of the Toluidine blue dye that interacts with mucin-derived sulphated glycoproteins in this region.

### IF staining: three-plex immuno-labelling and fluorescent imaging and analysis

The presence and density of NETs in colon tissue was visualised by using the Opal 6-Plex Detection Kit (NEL811001KT; AKOYA Biosciences) and simultaneously identifying MPO, CitH3 and NE in the same colon tissues using a multiplex imaging approach. All staining steps were completed at 22℃ in an opaque humidity chamber unless specified otherwise and following the manufacturer recommended protocol. After slide dewaxing and rehydrating, heat-induced epitope retrieval (HIER) was performed by placing the slides into an opaque Coplin jar with pH 6.0 citrate HIER buffer (S2369, DAKO) to expose the NETs antigens: MPO, NE and citH3. The device settings used for the HIER process and final dilutions were summarised in Supplementary Table S1. After HIER processing, slides were washed three times with tris-buffer saline (TBS) with 0.1% v/v Tween^®^ 20 (TBST) and one time with PBS to fully remove the HIER buffer. This was followed by incubation with 5% v/v H_2_O_2_ for 30 min to inhibit endogenous peroxidase activity and blocking with serum-free protein (X0909, DAKO) for 30 min to reduce non-specific antibody binding.

Next, slides were incubated with anti-NE primary antibody in antibody dilution buffer [1% w/v bovine serum albumin (BSA) and 0.5% v/v Triton-X100 in TBST) at 4℃ overnight. The following day, primary antibodies were removed by washing with 3 × 2 min TBST and 1 × 2 min PBS before incubating with Opal Polymer HRP Ms + Rb secondary antibodies for 45 min. Subsequently, colonic sections were incubated with Opal fluorophore (Opal 520) for 10 min in the dark. The HIER, primary antibody, secondary antibody and fluorophore incubation process was then repeated sequentially using anti-MPO and anti-citH3 antibodies with their respective fluorophores. Finally, triple-labelled colon slides were stained with 4′,6-diamidino-2-phenylindole (DAPI) to visualise cell nuclei and coverslipped with fluorescence mounting medium (S3023, DAKO). IF images were captured using an upright microscope (Axio Scope.A1, ZEISS) equipped with an AxioCam-ICm1 camera (ZEISS) at 10× objective. The imaging settings for different antibodies and their corresponding fluorophores are summarised in Supplementary Table S2.

The IF staining intensity of each NETs marker (MPO, NE and citH3) was analysed using the ImageJ Software (v.1.54d, National Institute of Health, U.S.A.). Briefly, multi-plex IF images were converted and channel separated using the Bio-Formats Plugin (v.7.3.0). Next, areas of the colon tissue as well as their corresponding cryptic areas were selected using the Freehand Selections tool in the ROI Manager. After area selection, staining intensity of each marker was then measured using the Multi Measure function in the ROI Manager tool (see Appendix S1 in Supplementary information). Overlap of the three immune markers, to identify NET density, was measured using ImageJ software (v.1.54d, National Institute of Health, U.S.A.) with the Trainable Weka Segmentation Plugin (v.3.3.4). To summarise, three representative images (with heavy, medium and light/no IF) were used to ‘train’ the AI plugin to accurately identify the presence of NETs. After verifying selectivity, the NETs selection criteria were applied to all images, and isolated NETs from each image file were identified in a new greyscale 8-bit image. The area of colon/crypts was highlighted using the Freehand Selections tool and stored in ROI manager, and the number of NETs was then determined using the Analyze Particles function (see Appendix S2 in Supplementary information).

### Tissue homogenisation for molecular and biochemical analysis

Isolated colon and faecal samples were homogenised to enable further molecular and biochemical analysis. Briefly, colon tissues or stool samples were snap frozen in liquid nitrogen then grounded into a fine powder with a mortar and pestle before resuspending with complete lysis buffer [50 mM phosphate buffer saline pH 7.4, 1 mM ethylenediaminetetraacetic acid, 10 µM butylated hydroxytoluene, 0.05 mM sodium azide, one tablet of cOmplete™ Protease Inhibitor Cocktail (Roche) and one tablet of PhosSTOP™ Phosphatase Inhibitor Cocktail (Roche)] in a 5 ml Teflon-coated tube (Wheaton Glassware). Next, the gross suspension was homogenised with a rotating piston matched to the Teflon-coated tube at 600 r.p.m. for 5 min whilst submerging in an ice bath, before centrifugation at 15,000x g for 15 min at 4℃. Clarified supernatants were collected, stored at −80℃ and total protein concentration was determined using the Pierce™ bicinchoninic acid (BCA) Protein Assay Kit (Thermo Scientific) by following the manufacturer recommended protocol.

#### Western blot assay

Western blot was used to visualise separated proteins in homogenised colon tissues using Precision Plus Protein™ Kaleidoscope™ (1610375, BioRad) ladder for molecular weight determination. Where required, 20 μg protein homogenised sample (except for 4-hydroxynonenal [4HNE], a marker of lipid oxidation that used 30 μg protein) was mixed with sodium dodecyl sulfate (SDS)-loading buffer (final concentration: 50 mM Tris-HCl pH 6.8, 2% w/v SDS, 6% v/v glycerol and 0.004% w/v bromophenol blue) and heat reduced at 95℃ for 5 min. After heating, samples were resolved on 12% w/v Mini-PROTEAN^®^ hand-cast gels (BioRad) in running buffer (containing: 25 mM Tris pH 8.3, 192 mM glycine and 0.1% w/v SDS) at 35 mA for 45 min using the Mini-PROTEAN^®^ chamber (BioRad).

Gels were then transferred onto 0.2 μm polyvinylidene fluoride (PVDF) membranes in transfer buffer (25 mM Tris pH 8.3, 192 mM glycine and 20% v/v methanol) using the Trans-Blot Turbo System (BioRad). After protein transfer, PVDF membranes were blocked with 5% w/v skim milk/TBST (1 h, 22℃) to minimise non-specific antibody binding. This is followed by the incubation with primary antibody anti-Nrf2, anti-GPx4, anti-SOD1 and anti-4HNE in antibody diluent buffer (5% w/v BSA, 0.05% w/v sodium azide in TBS) at 4℃ overnight. Anti-β actin visualisation of total β actin was used as a loading control. Subsequently, PVDF membranes were incubated with HRP-conjugated secondary antibodies [anti-rabbit HRP or anti-mouse HRP in 5% w/v skim milk in TBST for 2 h at 22°C (see Supplementary Table S3 for all antibodies dilutions). Protein bands of interest were visualised with Clarity™ Western ECL Substrate (1705060, BioRad) for 5 min at 22℃ in the dark after the membrane was washed 3 × 5 min with TBST and 1 × 5 min with TBS and images were captured using the ChemiDoc™ Touch Gel/Membrane Imaging System (BioRad). The densiometric intensity of the protein bands was analysed and normalised using the Image Lab software (v6.1, BioRad).

#### Enzyme linked immunosorbent assays and enzymatic activity assays

The following commercially available enzyme linked immunosorbent assay (ELISA) were purchased: Mouse IL-1β, IL-4, IL-10 and Calprotectin SimpleStep ELISA kit. Colorimetric enzymatic activity assay kits for colorimetric Activity Kit and SOD Activity Assay Kit were also obtained. The recommended protocols for manufacture were followed and clarified Colon or faecal homogenates were diluted with Mili-Q water to fit absorbance changes within the standard curve. The colorimetric absorbance of each sample was collected by using a microplate reader (Infinite M200-Pro, Tecan) (see Supplementary Table S3 for all sample dilution and microplate reader settings).

### Statistical analysis

All data collected in the current study were made available publicly via Mendely Data repository [74] and were subjected to heterogeneity (parametric) testing prior to group-wise comparison using GraphPad^®^ Prism software (v.10.1.0, La Jolla, U.S.A.). To minimise machine-introduced errors during the triple-plex IF analysis, statistical outliers were identified using the ROUT Method (*Q* = 1%) before normalcy testing and pair-wise comparisons were conducted. The heterogeneity and normality of the collected data were examined using the Shapiro–Wilk test with alpha (*α*) error = 0.05. For parametric data sets, one-way analysis of variance (ANOVA) test with Tukey’s multiple comparison as a post hoc analysis was conducted. The Kruskal–Wallis test with Dunn’s multiple comparison post hoc was used to identify any statistical differences between the treatment groups for non-parametrically distributed data sets. The statistical significance threshold between treatment groups was set as *P*<0.05, and all graphical data were represented as means ± standard deviation (SD).

## Supplementary material

Online supplementary material 1

## Data Availability

All data generated from this work are included in the manuscript and its supplementary files. All datasets generated for this study are available in a registered data repository (74).

## Abbreviations

ANOVA: Analysis of variance
AZD3241: MPO inhibitor
BSA: Bovine serum albumin
CAT: catalase
CD: Crohn’s disease
CP: Calprotectin
CPC: Charles Perkins Centre
CitH3: citrullinated histone H3
Cl^-^: Chloride anions
Clb: Blood clearance
DAI: Disease activity index
DAPI: 4′,6-diamidino-2-phenylindole
DPX: Dibutylphthalate plasticiser xylene
DSS: dextran sodium sulphate
ERK: MEK-extracellular-signal-regulated kinase
FCP: faecal calprotectin
GPR: G-protein-coupled receptor GPx
GPx1: glutathione peroxidase 1
GSK484: PAD4 inhibitor
H&E: Haematoxylin & eosin
5-HIAA: 5-hydroxyindoleacetic acid
4HNE: 4-hydroxynoneal
HO-1: heme oxygenase-1
H_2_O_2_: Hydrogen peroxide
IBD: inflammatory bowel disease
IF: Immunofluorescence
IL: Interleukin
IL-18R: Interleukin-18 receptor
MC: mast cells
MIT: Mast cell intracellular trap
MPO: myeloperoxidase
NE: neutrophil elastase
NETs: neutrophil extracellular traps
Nrf2: nuclear factor erythroid factor 2-related factor 2
PAD4: peptidyl-arginine deiminase IV
PB: Peanut butter
ROS: reactive oxygen species
SDS: Sodium dodecyl sulphate
SOD1: superoxide dismutase 1
TBS: Tris-buffered saline
TBST: Tris-buffered saline with Tween 20
TNF-α: Tumour necrosis factor alpha
UC: ulcerative colitis
