# Supplementary material for: The PAD4 inhibitor GSK484 diminishes neutrophil extracellular trap in the colon mucosa but fails to improve inflammatory biomarkers in experimental colitis
Source: Biosci Rep. 2025 Jun 11;45(6):375–97. doi: 10.1042/BSR20253205 (PMC12236107; doi:10.1042/BSR20253205)
Supplement: Online supplementary material 1 [file bcr-45-06-BSR20253205-supp1.docx]

**The PAD4 inhibitor GSK484 diminishes neutrophil extracellular trap in the colon mucosa but fails to improve inflammatory biomarkers in experimental colitis**

Kangzhe Xie^1,2^, Jordan Hunter^1,2,3^, Aaron Lee^1,2^, Gulfam Ahmad^1,4^, Paul K. Witting^1,2*^and Tamara Ortiz-Cerda^1,2,5*^

^1^ Redox Biology Group, School of Medical Sciences, Faculty of Medicine & Health, The University of Sydney, NSW 2006 Australia.

^2^ Charles Perkins Centre, School of Medical Sciences, Faculty of Medicine & Health, University of Sydney, Sydney, NSW 2006, Australia

^3^ Department of Biological Sciences, Purdue University, West Lafayette, Indiana, United States of America.

^4^ Andrology Department, Royal Women’s and Children’s Pathology, Carlton, VIC 3053, Australia.

^5^ Departmento de Citología e Histología Normal y patológica, Facultad de Medicina, Universidad de Sevilla, Spain. Avda. Sánchez-Pizjuán s/n 41009 Sevilla, Spain

^*^ Address correspondence to:

Dr Paul Witting, Level 4 West, The Charles Perkins Centre, The University of Sydney, Sydney, NSW 2006, Australia ([paul.witting@sydney.edu.au](mailto:paul.witting@sydney.edu.au))

Dr Tamara Ortiz-Cerda, Level 4 West, The Charles Perkins Centre, The University of Sydney, Sydney, NSW 2006, Australia ([tamara.ortizcerda@sydney.edu.au](mailto:tamara.ortizcerda@sydney.edu.au))

## Supplementary Tables & Figures

| **Antigen** | **Heat Induction Device** | **Retrieval Settings** | **Final dilution**  **(v/v)** | **Cat. number/**  **Supplier** |
| --- | --- | --- | --- | --- |
| MPO | Decloaking Chamber (Biocare Medical) | Pre-heat at 80℃ for 30sec and heat induced retrieval at 125℃ for 30sec; followed by cooling fan on at 95℃ and fan off at 90℃. | 1:100 | PA5-16672/ Invitrogen |
| NE | Microwave (Midea) | 1100W for 2min followed by 220W for 20min. | 1:500 | PA5-115648/ Invitrogen |
| CitH3 | Microwave (Midea) | 1100W for 2min followed by 220W for 20min. | 1:200 | ab219407/ abcam |

**Supplementary Table 1. Devices and settings used for heat-induced antigen retrieval for Immunofluorescence analysis.** MPO, Myeloperoxidase; NE, Neutrophil Elastase; CitH3, Citrullinated Histone H3.

| **Antigen** | **Opal Fluorophore** | **ZEISS Camera Filter Set** | **Beam Splitter** | **Excitation/Emission Wavelength** |
| --- | --- | --- | --- | --- |
| MPO | Opal 570 | Filter set 43 | FT570 | 550/570nm |
| NE | Opal 520 | Filter set 44 | FT500 | 494/525nm |
| CitH3 | Opal 690 | Filter set 50 | FT660 | 676/694nm |
| - | DAPI | Filter set 49 | FT395 | 358/461nm |

**Supplementary Table 2.** **Imaging settings for neutrophil extracellular trap markers for immunofluorescence analysis.** MPO, Myeloperoxidase; NE, Neutrophil Elastase; CitH3, Citrullinated Histone H3; DAPI, 4′,6-diamidino-2-phenylindole.

|  | **Protein**  **analysis** | **Optimised Dilution**  **(v/v)** | **Cat. number/**  **Supplier** | **Wavelength (nm)** |
| --- | --- | --- | --- | --- |
| **Antigen** | Nrf2 | 1:2000 | PA5-88084, Invitrogen | - |
|  | GPx4 | 1:1000 | ab125066, abcam |  |
|  | SOD1 | 1:2000 | SAB5200083, Sigma Aldrich |  |
|  | 4HNE | 1:800 | BS-6313R, Bioss |  |
|  | β actin | 1:10000 | 4967S, Cell Signaling Technology |  |
|  | Rabbit HRP | 1:2000 | A6154, Sigma Aldrich |  |
|  | Mouse HRP | 1:2000 | ab205719, abcam |  |
| **Kits** | IL-1β ELISA | 1:50 | BMS6002, Invitrogen | 450 |
|  | IL-4 ELISA | 1:20 | BMS613, Invitrogen | 450 |
|  | IL-10 ELISA | 1:100 | BMS614, Invitrogen | 450 |
|  | Calprotectin (Stool) ELISA | 1:20 | ab263885, Abcam | 450 |
|  | Calprotectin (Colon) ELISA | 1:100 | ab263885, Abcam | 450 |
|  | CAT Activity | 1:4 | EIACATC, Invitrogen | 560 |
|  | SOD Activity | No Dilution Performed | ab65354, Abcam | 450 |

**Supplementary Table 3.** **Sample dilution factor used for different Western blot analysis, enzyme linked immunosorbent assay (ELISA) and enzymatic activity assay kits.** Nrf2, anti-nuclear factor erythroid 2-related factor 2; GPx4, Glutathione peroxidase 4; SOD1, Superoxide dismutase-1; HRP, Horseradish peroxidase; IL, Interleukin; CAT, Catalase; SOD, Superoxide Dismutase.


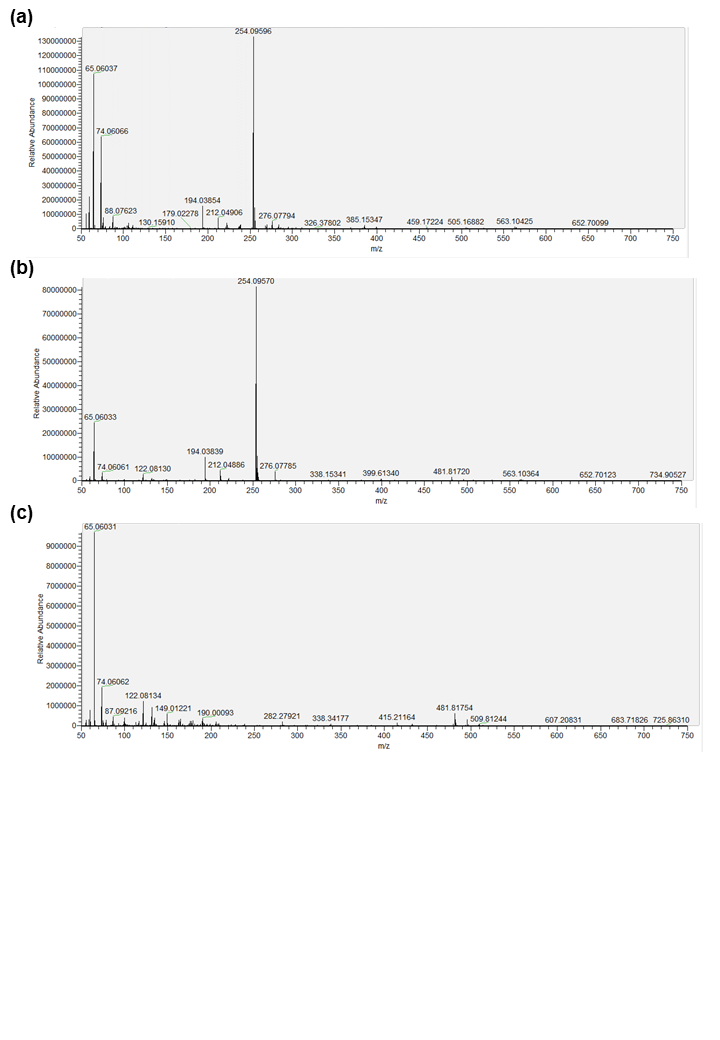


**Supplementary Figure 1.** **Electrospray mass spectrometry of AZD3241 showed impurities in the inhibitor supplied by MedChemExpress.** **(a)** Representative abundance peaks of AZD3241 from MedChemExpress at 5 µg/mL in 50% v/v methanol. **(b)** Representative abundance peaks of AZD3241 from Pharmaxis at 5 µg/mL in 50% v/v methanol. **(c)** Representative abundance peaks of 50% v/v methanol as a blank.


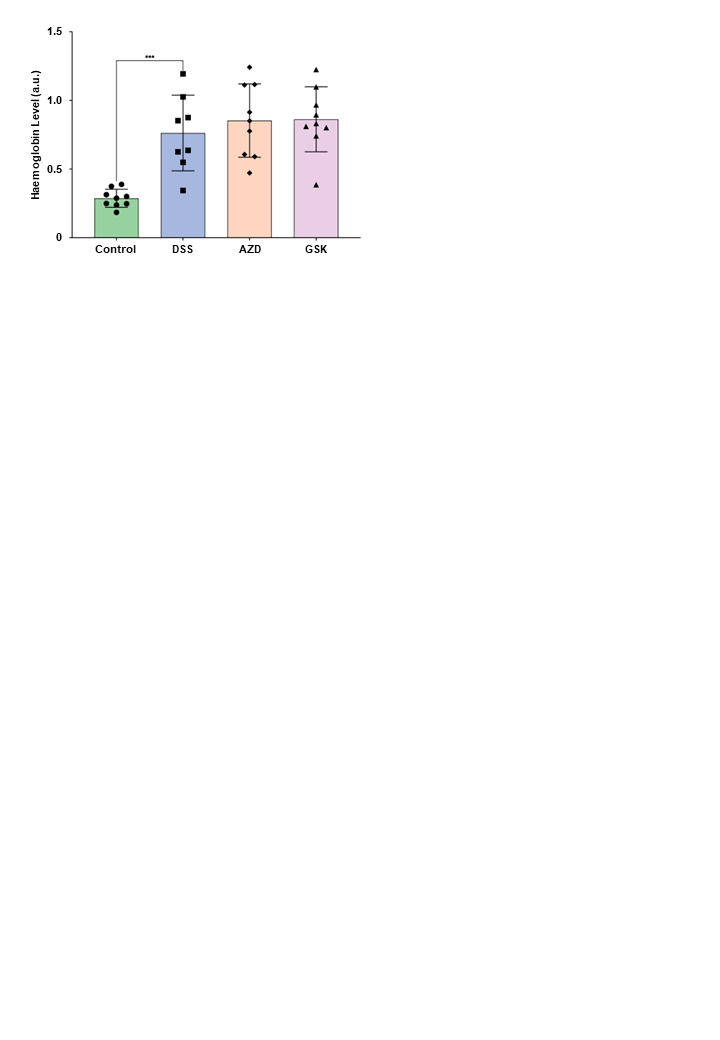


**Supplementary Figure 2.** **The effect of MPO and/or PAD4 inhibition on faecal haemoglobin level**. Graphical values represent mean ± SD with n=9 mice per group. Protocol for faecal haemoglobin followed the assay described in **Appendix 3**. Normalcy of the collected data was analysed using Shapiro-Wilk test, group-wise comparison was performed by using one way ANOVA with Tukey’s multiple comparison as a post hoc test. * p ≤ 0.05, ** p ≤ 0.01, *** p ≤ 0.001 and **** p ≤ 0.0001.


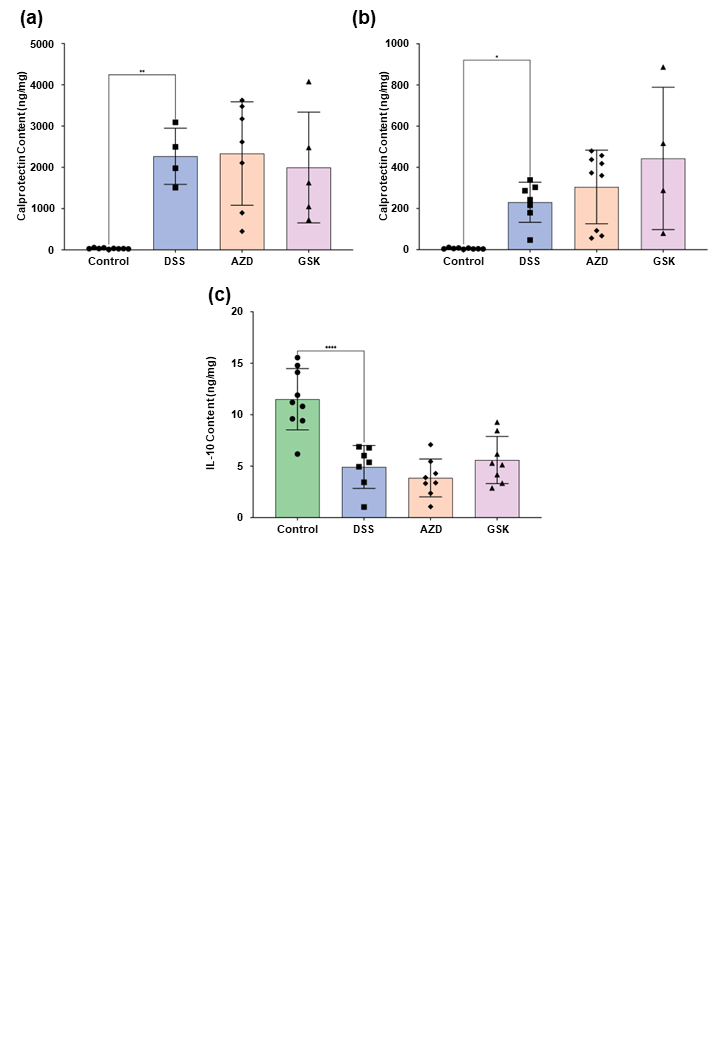


**Supplementary Figure 3.** **The effect of MPO and/or PAD4 inhibition on colon inflammatory markers.** **(a)** Colon calprotectin. **(b)** FCP, Faecal Calprotectin Stool calprotectin. Graphical values represent mean ± SD with n = 4-9 mice per group after standard curve interpolation. Normalcy of the collected data was analysed using Shapiro-Wilk test, group difference was analysed by one way ANOVA with Tukey’s multiple comparison for parametric data and Kruskal-Wallis test with Dunn’s multiple comparison test was used for non-parametric data. * p ≤ 0.05, ** p ≤ 0.01, *** p ≤ 0.001 and **** p ≤ 0.0001.


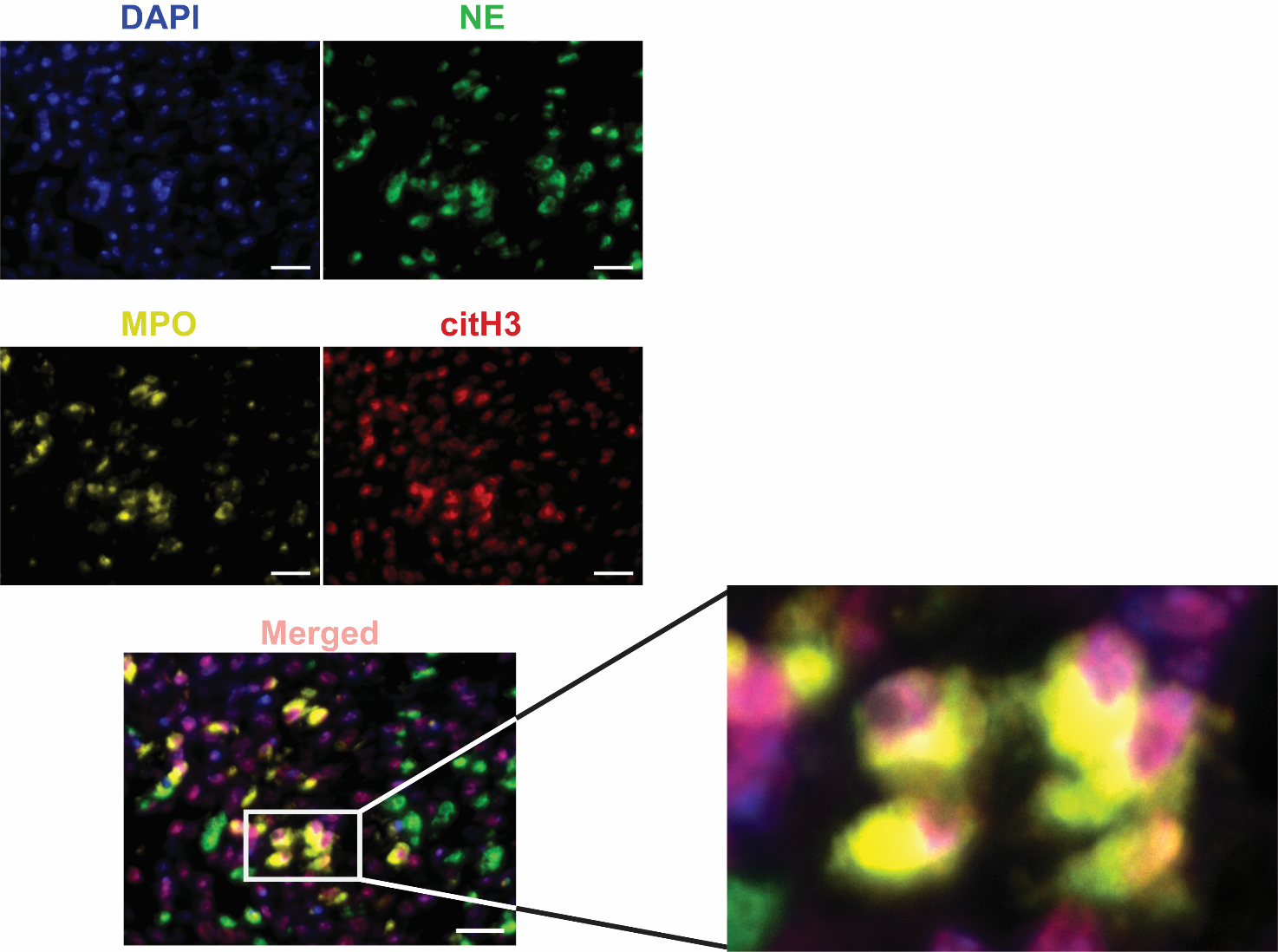


**Supplementary Figure 4. Representative images of triple-plex immunofluorescence images of colon NETs.** Expanded view shows immune^+^ signal from NE (Green), MPO (yellow) and CitH3 (red) and is indicative of the spatial colocalization of the three proteins that are critical to the formation of NETs in the extracellular domain. Images were captured using an Axio Scope.A1 fluorescence microscope with a AxioCam-ICm1 camera at 10x magnification. Scale bar = 100 µm.


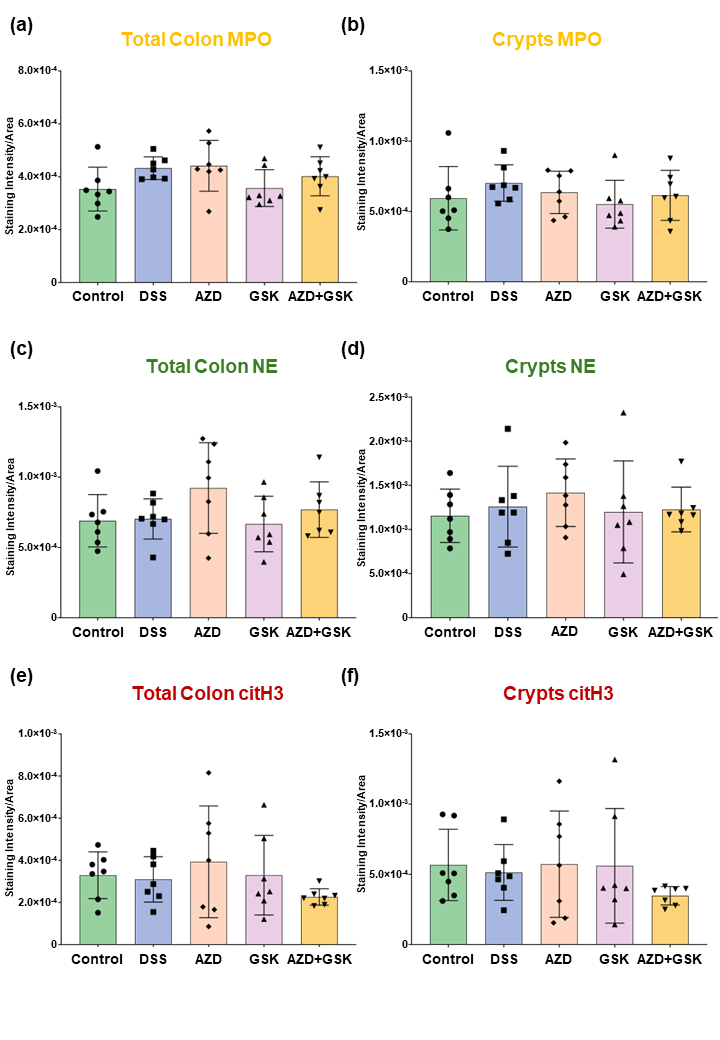


**Supplementary Figure 5. Immunofluorescence staining intensity of MPO, NE and citH3 in mouse colons with/without DSS insults. (a)** Total MPO staining intensity in the colon. **(b)** MPO staining intensity in the cryptic region only. (c). Total NE staining intensity in the colon. (d). NE staining intensity in the cryptic region only. **(e)** Total citH3 staining intensity in the colon. **(f)** citH3 staining intensity in the cryptic region only. Graphical values represent mean ± SD with n = 7 mice per group. Statistical outliers were identified and removed using the ROUT method (Q = 1%) and data normality was tested using the Shapiro-Wilk test. Group-wise comparison was performed by using one way ANOVA with Tukey’s multiple comparison as a post hoc test.


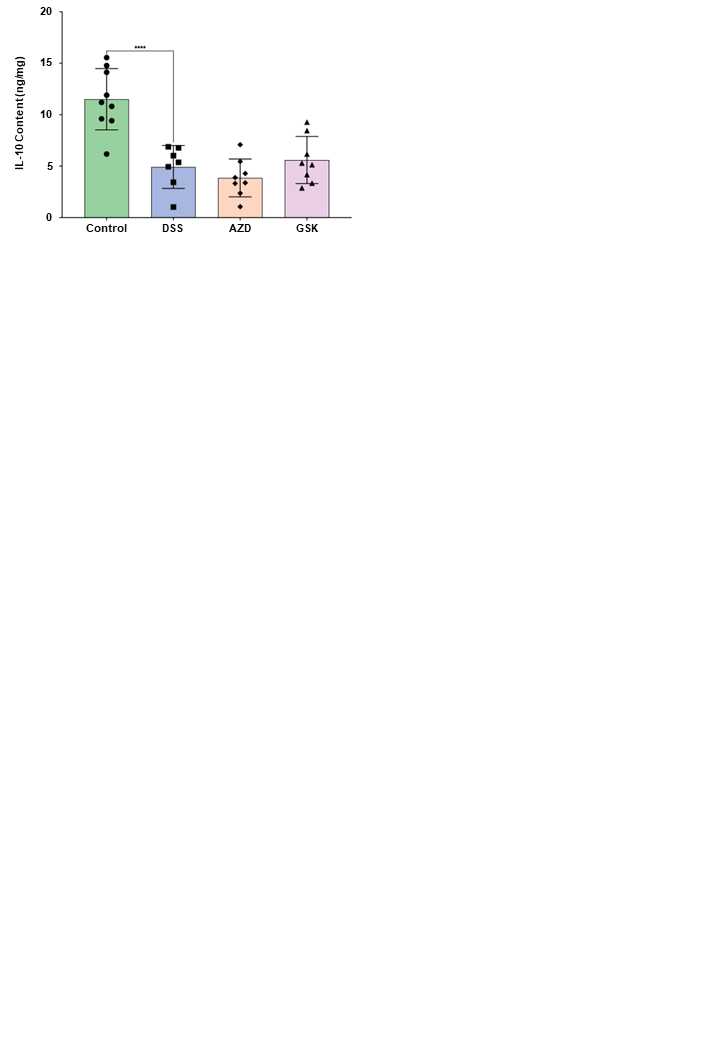


**Supplementary Figure 6. The effect of MPO and/or PAD4 inhibition on colon IL-10 levels.** Graphical values represent mean ± SD with n = 7-9 mice per group after standard curve interpolation. Normalcy of the collect data was analysed using Shapiro-Wilk test, group difference was analysed by one way ANOVA with Tukey’s multiple comparison. * p ≤ 0.05, ** p ≤ 0.01, *** p ≤ 0.001 and **** p ≤ 0.0001.

## Supplementary Information

**Appendix 1.** Macro code for NETs staining intensity analysis.

dir1=getDirectory("");

list=getFileList(dir1);

setBatchMode("show");

for (i=0; i<list.length; i++) {showProgress(i+1, list.length);

open(dir1+list[i]);

fileName = getInfo("image.filename");

selectWindow(fileName + " - C=0");

selectWindow(fileName + " - C=1");

selectWindow(fileName + " - C=2");

selectWindow(fileName + " - C=3");

run("ROI Manager...");

//setTool("freehand");

selectWindow(fileName + " - C=0");

waitForUser("Select Total Colon Area Now");

roiManager("Add");

roiManager("Select", 0);

roiManager("Rename", "Total");

waitForUser("Select Crypts Area Now");

roiManager("Add");

roiManager("Select", 1);

roiManager("Rename", "Crypts");

run("Tile");

selectWindow(fileName + " - C=1");

roiManager("Show All");

selectWindow(fileName + " - C=2");

roiManager("Show All");

selectWindow(fileName + " - C=3");

roiManager("Show All");

roiManager("Deselect");

selectWindow(fileName + " - C=1");

roiManager("multi-measure measure_all one append");

selectWindow(fileName + " - C=2");

roiManager("multi-measure measure_all one append");

selectWindow(fileName + " - C=3");

roiManager("multi-measure measure_all one append");

selectWindow(fileName + " - C=0");

close();

selectWindow(fileName + " - C=1");

close();

selectWindow(fileName + " - C=2");

close();

selectWindow(fileName + " - C=3");

roiManager("Select", 0);

roiManager("Delete");

roiManager("Select", 0);

roiManager("Delete");

close();}

**Appendix 2.** Macro code used for NETs counting analysis.

dir1=getDirectory("");

list1=getFileList(dir1);

dir2=getDirectory("");

list2=getFileList(dir2);

setBatchMode("show");

for (i=0; i<list1.length; i++) {showProgress(i+1, list1.length);

for (i=0; i<list2.length; i++) {showProgress(i+1, list2.length);

open(dir1+list1[i]);

fileName1 = getInfo("image.filename");

open(dir2+list2[i]);

fileName2 = getInfo("image.filename");

selectWindow(fileName2);

setAutoThreshold("Default no-reset");

//run("Threshold...");

setThreshold(0, 0, "raw");

//setThreshold(0, 0);

setOption("BlackBackground", true);

run("Convert to Mask");

selectWindow(fileName1);

//setTool("freehand");

run("ROI Manager...");

waitForUser("Select Total Colon Area Now");

roiManager("Add");

roiManager("Select", 0);

roiManager("Rename", "Total");

waitForUser("Select Crypts Area Now");

roiManager("Add");

roiManager("Select", 1);

roiManager("Rename", "Crypts");

selectWindow(fileName2);

roiManager("Select", 0);

run("Analyze Particles...", "summarize");

wait(3000);

roiManager("Show None");

roiManager("Select", 1);

run("Analyze Particles...", "summarize");

wait(3000);

roiManager("Select", 0);

roiManager("Delete");

roiManager("Select", 0);

roiManager("Delete");

selectWindow(fileName1);

close();

selectWindow(fileName2);

close();}

**Appendix 3.** Protocol for faecal haemoglobin analysis.

The level of haemoglobin in the stool homogenates was analysed using a colorimetric assay that was previously published by our group (See Fecal HB and Calprotectin Analysis in the METHODS section of Ref^1^). Briefly, 200µL of thawed stool homogenates were loaded onto a 96-well assay plate in duplicates (Greiner Bio-One). The absorbance reading of each sample was measured at 402nm by using microplate reader (Infinite M200-Pro, Tecan).

**Appendix 4.** Full membrane images of Nrf2 Western blotting.


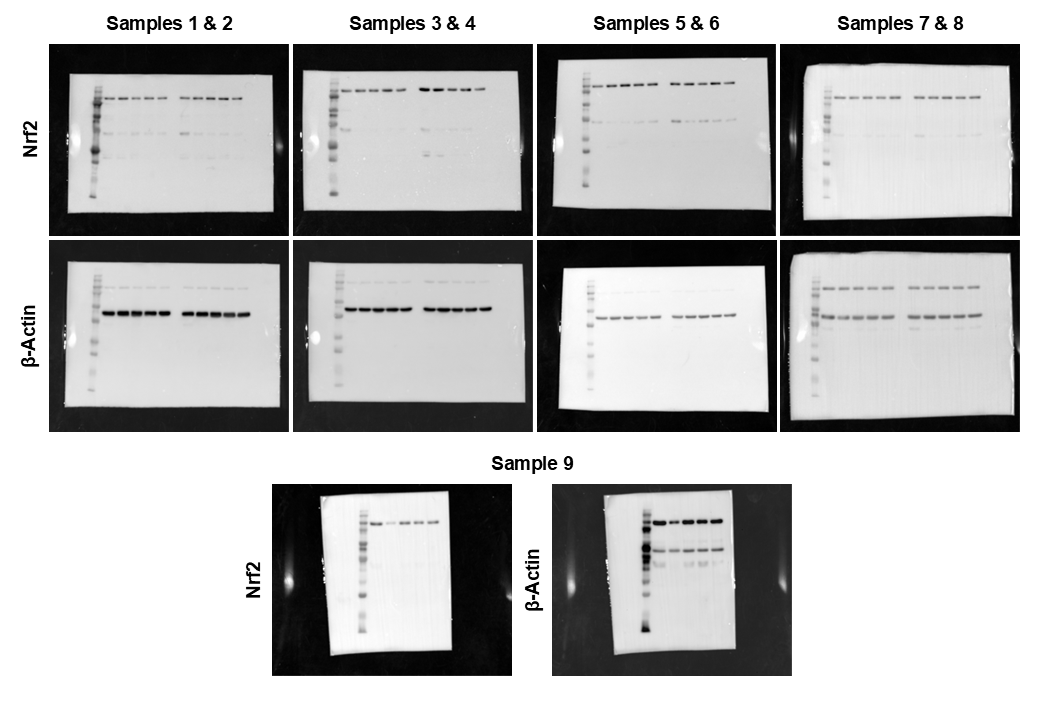


**Appendix 5.** Full membrane images of GPx4 Western blotting.


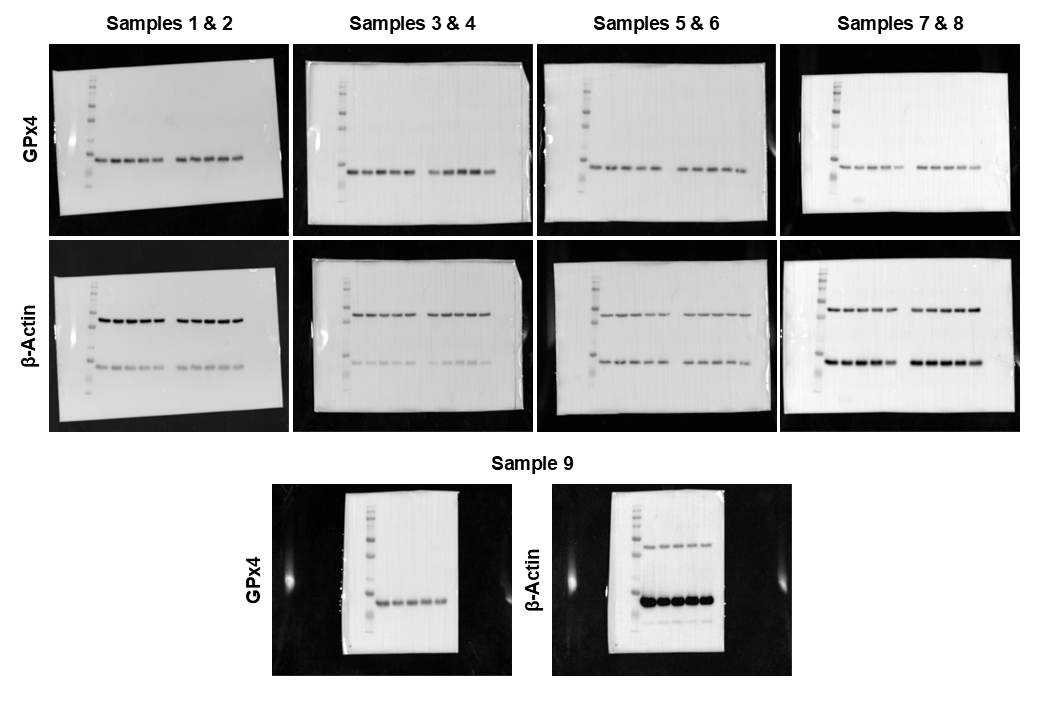


**Appendix 6.** Full membrane images of SOD1 Western blotting.


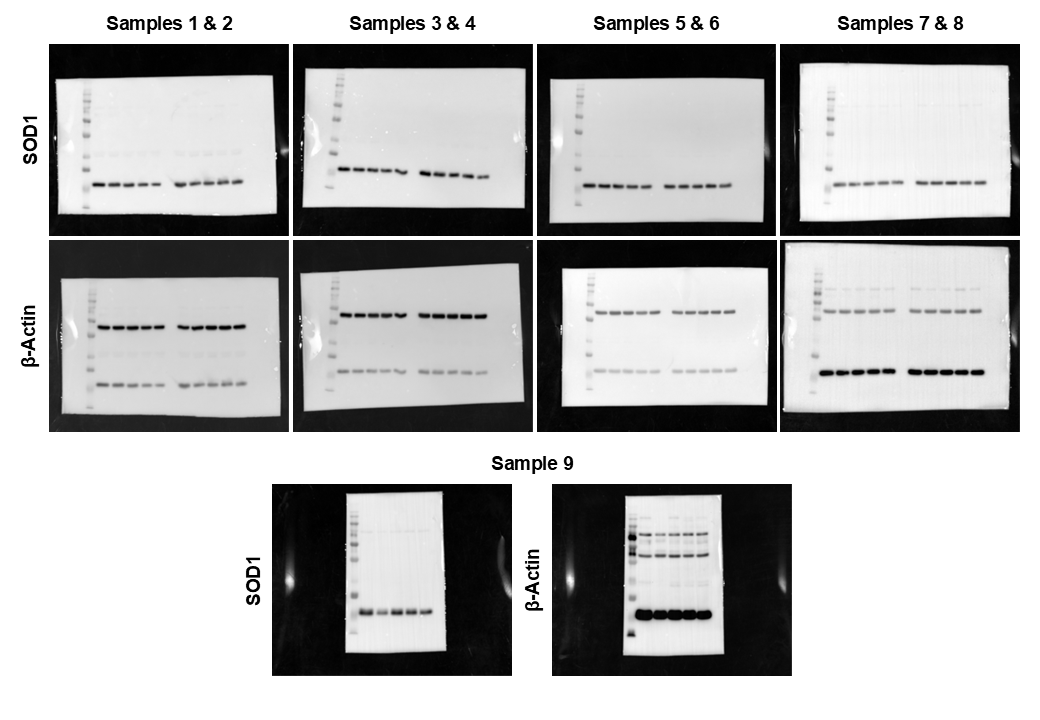


**Appendix 7.** Full membrane images of 4HNE Western blotting.


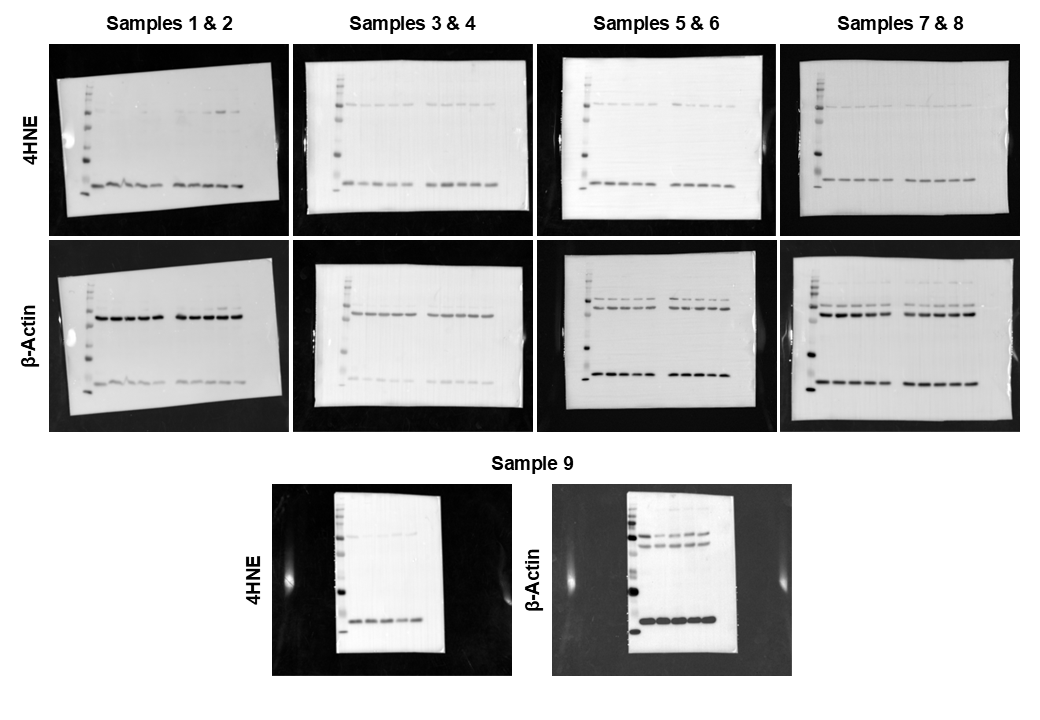


## References for Supplementary Information

[1] Ahmad G, Chami B, Liu Y, Schroder AL, San Gabriel PT, Gao A, Fong G, Wang X, Witting PK: The Synthetic Myeloperoxidase Inhibitor AZD3241 Ameliorates Dextran Sodium Sulfate Stimulated Experimental Colitis. Front Pharmacol 2020, 11:556020.
